# Firefly toxin lucibufagins evolved after the origin of bioluminescence

**DOI:** 10.1093/pnasnexus/pgae215

**Published:** 2024-06-25

**Authors:** Chengqi Zhu, Xiaoli Lu, Tianlong Cai, Kangli Zhu, Lina Shi, Yinjuan Chen, Tianyu Wang, Yaoming Yang, Dandan Tu, Qi Fu, Jing Huang, Ying Zhen

**Affiliations:** Westlake Laboratory of Life Sciences and Biomedicine, Hangzhou, Zhejiang 310024, China; Key Laboratory of Structural Biology of Zhejiang Province, School of Life Sciences and Research Center for Industries of the Future, Westlake University, Hangzhou, Zhejiang 310024, China; Institute of Biology, Westlake Institute for Advanced Study, Hangzhou, Zhejiang 310024, China; Westlake Laboratory of Life Sciences and Biomedicine, Hangzhou, Zhejiang 310024, China; Key Laboratory of Structural Biology of Zhejiang Province, School of Life Sciences and Research Center for Industries of the Future, Westlake University, Hangzhou, Zhejiang 310024, China; Institute of Biology, Westlake Institute for Advanced Study, Hangzhou, Zhejiang 310024, China; Westlake Laboratory of Life Sciences and Biomedicine, Hangzhou, Zhejiang 310024, China; Westlake Laboratory of Life Sciences and Biomedicine, Hangzhou, Zhejiang 310024, China; Westlake Laboratory of Life Sciences and Biomedicine, Hangzhou, Zhejiang 310024, China; Key Laboratory of Structural Biology of Zhejiang Province, School of Life Sciences and Research Center for Industries of the Future, Westlake University, Hangzhou, Zhejiang 310024, China; Institute of Biology, Westlake Institute for Advanced Study, Hangzhou, Zhejiang 310024, China; Instrumentation and Service Center for Molecular Sciences, Westlake University, Hangzhou, Zhejiang 310030, China; Westlake Laboratory of Life Sciences and Biomedicine, Hangzhou, Zhejiang 310024, China; Key Laboratory of Structural Biology of Zhejiang Province, School of Life Sciences and Research Center for Industries of the Future, Westlake University, Hangzhou, Zhejiang 310024, China; Institute of Biology, Westlake Institute for Advanced Study, Hangzhou, Zhejiang 310024, China; Westlake Laboratory of Life Sciences and Biomedicine, Hangzhou, Zhejiang 310024, China; Key Laboratory of Structural Biology of Zhejiang Province, School of Life Sciences and Research Center for Industries of the Future, Westlake University, Hangzhou, Zhejiang 310024, China; Institute of Biology, Westlake Institute for Advanced Study, Hangzhou, Zhejiang 310024, China; Westlake Laboratory of Life Sciences and Biomedicine, Hangzhou, Zhejiang 310024, China; Key Laboratory of Structural Biology of Zhejiang Province, School of Life Sciences and Research Center for Industries of the Future, Westlake University, Hangzhou, Zhejiang 310024, China; Institute of Biology, Westlake Institute for Advanced Study, Hangzhou, Zhejiang 310024, China; Westlake Laboratory of Life Sciences and Biomedicine, Hangzhou, Zhejiang 310024, China; Key Laboratory of Structural Biology of Zhejiang Province, School of Life Sciences and Research Center for Industries of the Future, Westlake University, Hangzhou, Zhejiang 310024, China; Institute of Biology, Westlake Institute for Advanced Study, Hangzhou, Zhejiang 310024, China; Westlake Laboratory of Life Sciences and Biomedicine, Hangzhou, Zhejiang 310024, China; Key Laboratory of Structural Biology of Zhejiang Province, School of Life Sciences and Research Center for Industries of the Future, Westlake University, Hangzhou, Zhejiang 310024, China; Institute of Biology, Westlake Institute for Advanced Study, Hangzhou, Zhejiang 310024, China; Westlake Laboratory of Life Sciences and Biomedicine, Hangzhou, Zhejiang 310024, China; Key Laboratory of Structural Biology of Zhejiang Province, School of Life Sciences and Research Center for Industries of the Future, Westlake University, Hangzhou, Zhejiang 310024, China; Institute of Biology, Westlake Institute for Advanced Study, Hangzhou, Zhejiang 310024, China

**Keywords:** firefly, lucibufagin, Na^+^, K^+^-ATPase, ATPα, LBG

## Abstract

Fireflies were believed to originally evolve their novel bioluminescence as warning signals to advertise their toxicity to predators, which was later adopted in adult mating. Although the evolution of bioluminescence has been investigated extensively, the warning signal hypothesis of its origin has not been tested. In this study, we test this hypothesis by systematically determining the presence or absence of firefly toxin lucibufagins (LBGs) across firefly species and inferring the time of origin of LBGs. We confirm the presence of LBGs in the subfamily Lampyrinae, but more importantly, we reveal the absence of LBGs in other lineages, including the subfamilies of Luciolinae, Ototretinae, and Psilocladinae, two *incertae sedis* lineages, and the Rhagophthalmidae family. Ancestral state reconstructions for LBGs based on firefly phylogeny constructed using genomic data suggest that the presence of LBGs in the common ancestor of the Lampyrinae subfamily is highly supported but unsupported in more ancient nodes, including firefly common ancestors. Our results suggest that firefly LBGs probably evolved much later than the evolution of bioluminescence. We thus conclude that firefly bioluminescence did not originally evolve as direct warning signals for toxic LBGs and advise that future studies should focus on other hypotheses. Moreover, LBG toxins are known to directly target and inhibit the α subunit of Na^+^, K^+^-ATPase (ATPα). We further examine the effects of amino acid substitutions in firefly ATPα on its interactions with LBGs. We find that ATPα in LBG-containing fireflies is relatively insensitive to LBGs, which suggests that target-site insensitivity contributes to LBG-containing fireflies' ability to deal with their own toxins.

**Significance Statement**

Why did fireflies originally evolve bioluminescence? A popular hypothesis is that bioluminescence first evolved as warning signals to firefly toxin lucibufagins (LBGs) and was later repurposed as adult mating signals. Based on this hypothesis, LBGs should have evolved at the same time or earlier than firefly bioluminescence. In this study, we examined the evolution of LBG toxins based on a confident firefly species tree. Surprisingly, we found that LBGs probably evolved once in only one subgroup of fireflies, much later than the origin of bioluminescence, implying that bioluminescence did not originate as warning signals for toxic LBGs. Based on the time of origin and reported antioxidant effects of luciferin, we hypothesized that firefly bioluminescence might have originally evolved to deal with the increasing oxidative stress.

## Introduction

Understanding the origin of novel traits is a central goal in evolutionary biology. However, the task could be challenging due to the unknown ecological context at the time of origin and the potential modification of the trait's function over time. Bioluminescence, as one of the most captivating novel traits, has evolved nearly 100 times among diverse organisms on earth (1, 2). Bioluminescence serves diverse biological functions across species, including aposematism (3, 4), coping with oxidative stress (5), and interspecies or intraspecies communication (6–8). Fireflies are the most common and one of the few bioluminescent terrestrial organisms (9, 10). Fireflies are commonly referred to as the Lampyridae family of beetles, in which eight subfamilies are currently recognized, namely Lampyrinae, Luciolinae, Pterotinae, Ototretinae, Lamprohizinae, Psilocladinae, Amydetinae, and Photurinae. Together with the sister lineages of Rhagophthalmidae and Phengodidae families, this represents one of three known beetle lineages that parallelly evolved bioluminescence (10, 11).

While the most well-known function of bioluminescence in fireflies is adult communication, bioluminescence is also present in the immature stages of fireflies, including eggs, larvae, and pupae (12). The conspicuous glowing at relatively nonmobile or less mobile immature stages (13), and the fact that some firefly species possess noxious toxins, suggest that bioluminescence in fireflies may have initially evolved as a warning signal for their toxins across developmental stages and later repurposed for adult communications (12, 14). However, to date, this intriguing hypothesis has not been formally tested.

Several studies have experimentally examined the role of firefly bioluminescence as an aposematic display (3, 15). These studies have found that potential predators, such as house mice and jumping spiders, reject noxious fireflies and learn to avoid bitter food with flashing LEDs faster than without light cues (4, 16). Additionally, nocturnal predator big brown bats have been shown to learn to avoid *Photinus pyralis* fireflies by integrating information from multiple sensors, including visual bioluminescence signals (17). Toads collected from glow-worm habitat have also been reported to reduce their attacks to luminescent artificial prey (18). These findings suggest that firefly bioluminescence serves as a warning signal for predators to avoid noxious prey.

Many firefly species were found to be distasteful to predators because they are chemically defended (19–24). The defensive substances were first isolated from North American species of *Photinus ignitus* and *Photinus marginellus* and named lucibufagins (LBGs), which were apparently produced by fireflies themselves from dietary steroids (11, 22, 24). LBGs were subsequently identified from several firefly species in the genera *Lucidota*, *Ellychnia*, and *Lampyris* (14, 24, 25). A recent study detected LBGs in 21 species from 4 genera of Lampyrinae in European fireflies using museum specimens (26). LBGs are the only group of toxins that have been reported in more than one firefly species, despite other defensive compounds such as *N*-methylquinolinium 2-carboxylate and terpinolene also being reported in specific firefly species (27, 28). LBGs belong to a subclass of cardiotonic steroids (CTSs), which include structurally similar cardenolides and bufadienolides that are defensive chemicals in Apocynaceae plants and toxic toads, respectively (25, 29). CTSs are toxic to most animals because they directly bind to and inhibit the ion transport ability of ATPα, the alpha subunit of the Na^+^, K^+^-ATPase (NKA) (30, 31). ATPα is highly conserved across animals and plays essential roles in fundamental biological processes such as muscle contraction, osmoregulation, and neural signal transduction (32). There are also some firefly species that lack LBGs. It has been reported that *Photuris* species prey on and sequester LBGs from toxic *Photinus* species, although they cannot synthesize LBGs *de novo* (23, 33). Additionally, LBGs have not been found in *Aquatica lateralis* (11), multiple species from *Luciola* genus in Luciolinae, as well as two genera in Lamprohizinae (26).

These findings raise the question of whether LBGs co-evolved with bioluminescence in the common ancestors of all fireflies and were subsequently lost in some lineages or whether LBGs evolved after the evolution of bioluminescence within a subgroup of fireflies. If firefly bioluminescence evolved as the conspicuous warning signal of firefly LBG toxins, we would expect that LBG biosynthesis in fireflies evolved at the same time or earlier than the evolution of firefly bioluminescence (34, 35). Recent studies have traced the evolution of bioluminescence in Elateroidea beetles and found that bioluminescence in the Lampyridae lineage evolved before the common ancestor of fireflies (10, 11, 36, 37). The oldest firefly fossil dated back to mid-Cretaceous, suggesting that the common ancestor of Lampyridae existed even before this (38, 39).

A confident species tree is essential for inferring the origin and evolution of LBGs in fireflies. Martin et al. (40) employed an anchored hybrid enrichment approach to obtain genome-level sequence information from 88 species in 53 genera from 8 subfamilies. They reported that none of the previously recognized subfamilies were recovered as monophyletic, leading to extensive revisions to previous phylogenies based on morphological characters and a small number of genetic markers (40). However, due to the extremely limited geographic and temporal distributions of many firefly species and the challenges involved in acquiring samples in suitable conditions to collect genomic data, only five Lampyridae species and one click beetle species have both genome-level data for phylogenetic relationships and information on the presence or absence of LBGs (26, 40). Therefore, the small number of species with both LBGs and confirmed phylogenetic relationships precludes the inference of LBG evolution.

In this study, we investigated the evolution of LBG toxins in fireflies. We systematically surveyed LBGs across 21 representative species, including 19 firefly species from 12 genera of Lampyridae, and 1 species from each of the outgroup families Rhagophthalmidae and Cantharidae. We conducted an extensive search for the presence of LBGs in these species using liquid chromatography–mass spectrometry (LC–MS). Moreover, we constructed a species tree using genomic and transcriptome data and performed ancestral state reconstruction to infer the origin of LBGs in fireflies. Lastly, we examined whether ATPα from LBG-containing fireflies had amino acid substitutions that could contribute to insensitivity to LBG toxins.

## Results

### A confident firefly phylogeny based on genome-wide data

**Fig. 1.**
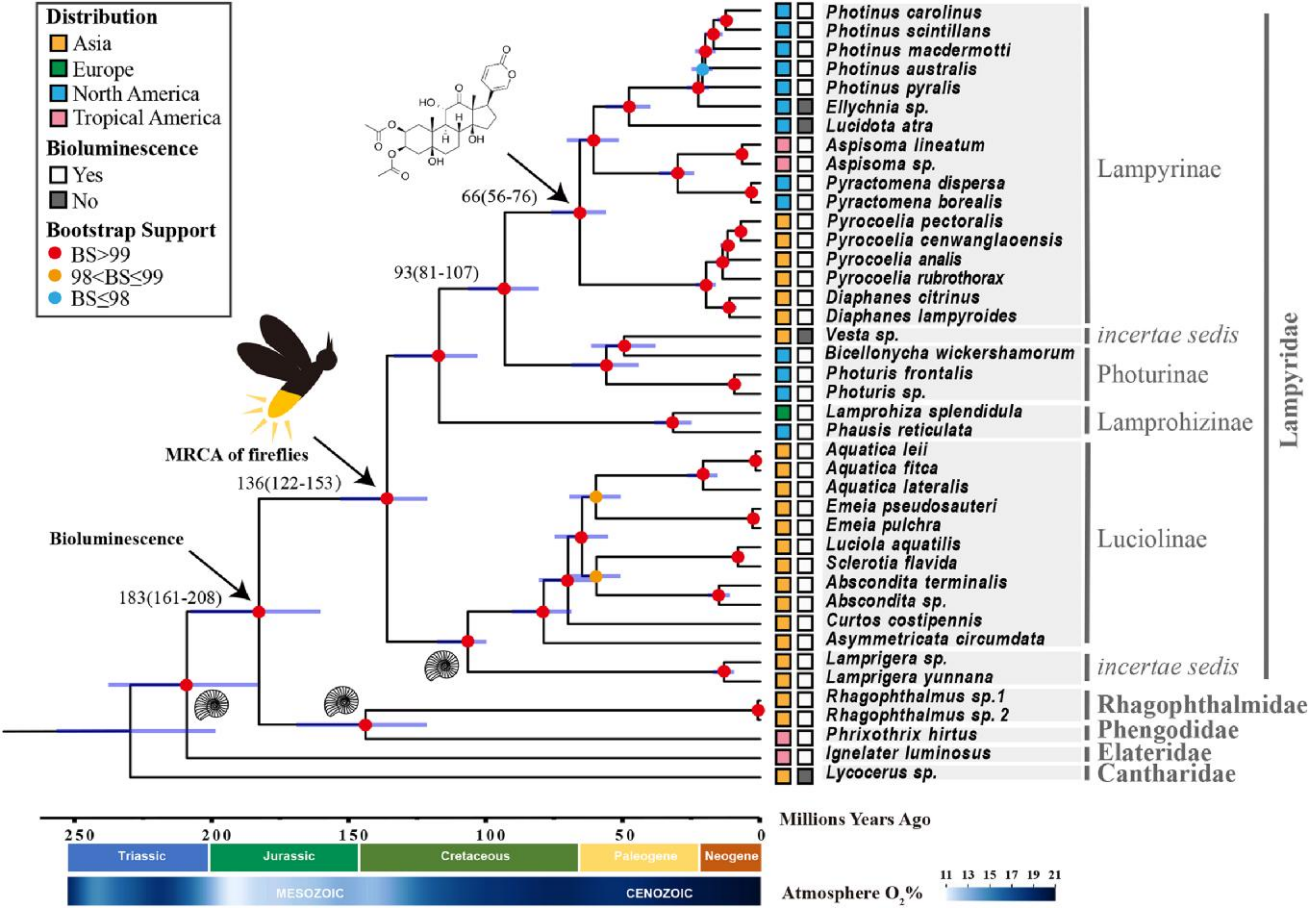
A time-calibrated ML phylogeny of 41 beetles based on 1,353 ortholog nucleotide sequences. The node when firefly LBGs first evolved is marked by an LBG molecule and the MRCAs of Lampyridae are marked with a firefly cartoon. The horizontal bars over the nodes show 95% HPD intervals of divergence time estimated from MCMCTree. The numbers over the nodes are the estimated median divergence times in Mya with 95% HPD intervals. The fossil cartoons on the nodes represent the three fossil calibrations used. The color bar located at the bottom shows historical levels of oxygen (43).

Due to the limited geographic and temporal distributions of firefly species, there is a scarcity of species for which both LBG information and genomic data are available. To establish a confident phylogeny for inferring the origin and evolution of LBGs in fireflies, we collected fresh samples from 16 species from the Lampyridae family, including 3 newly identified species (41, 42), and 1 species from each of the outgroup families Rhagophthalmidae and Cantharidae (Table S1). Eight firefly subfamilies were currently recognized, and three additional lineages were placed as *incertae sedis* in Lampyridae (40). Samples were identified to species or genus based on morphological and behavioral characteristics, as well as CO1 barcoding sequences when available from BOLD and NCBI databases (Table S1). We sequenced the transcriptomes of 16 of these species that did not have publicly available genomic data. Together with other publicly available RNA-seq data, we *de novo* assembled transcriptomes of 35 firefly species. Combined with 6 species that had whole genomes sequenced, we compiled genomic level data from 41 species, with a focus on Lampyridae (Lampyrinae: 7 genera, Lamprohizinae: 2 genera, Luciolinae: 7 genera, Photurinae: 2 genera; 2 *incertae sedis* lineages: *Vesta* and *Lamprigera*), representatives from each of the 4 bioluminescence beetle families and 1 outgroup species (Fig. 1; Tables S2 and S3).

Using this dataset, we identified 1,353 single-copy orthologs, allowing 20% missingness across 41 species, with a total length of 1,425,789 bp after filtering. Phylogenetic trees were constructed based on concatenated nucleotide or protein sequences of these genes using the maximum likelihood method, and rooted by the nonbioluminescent *Lycocerus* sp. from Cantharidae family. The majority of the nodes are highly supported for both DNA and protein trees, and consistent with each other (Fig. S1). Rhagophthalmidae and Phengodidae form a sister group to Lampyridae, and the bioluminescent click beetle *Ignelater luminosus* is the outgroup of all these families. Furthermore, we recovered the major subdivisions within Lampyridae, including Lampyrinae, Photurinae, Lamprohizinae, and Luciolinae. Consistent with recent studies using genome and mitogenome data, our results support the phylogenetic relationships of two Lampyridae *incertae sedis* lineages noted by Martin et al., that *Vesta* sp. clusters with two Photurinae genera *Bicellonycha* and *Photuris*, and that *Lamprigera* is a sister clade to Luciolinae rather than a member of Lampyrinae (40, 44). The results also support Martin et al. on the removal of Lamprohizini tribe from Lampyrinae subfamily, and Lamprohizini being elevated to Lamprohizinae subfamily as a sister group to Lampyrinae and Photurinae subfamilies (Figs. 1 and S1) (40, 45). This confident firefly phylogeny based on genome data, coupled with species-specific LBG information we obtained, enables us to infer the evolution of LBG toxins in fireflies.

### LBG screening using LC–MS across fireflies and outgroups

**Fig. 2.**
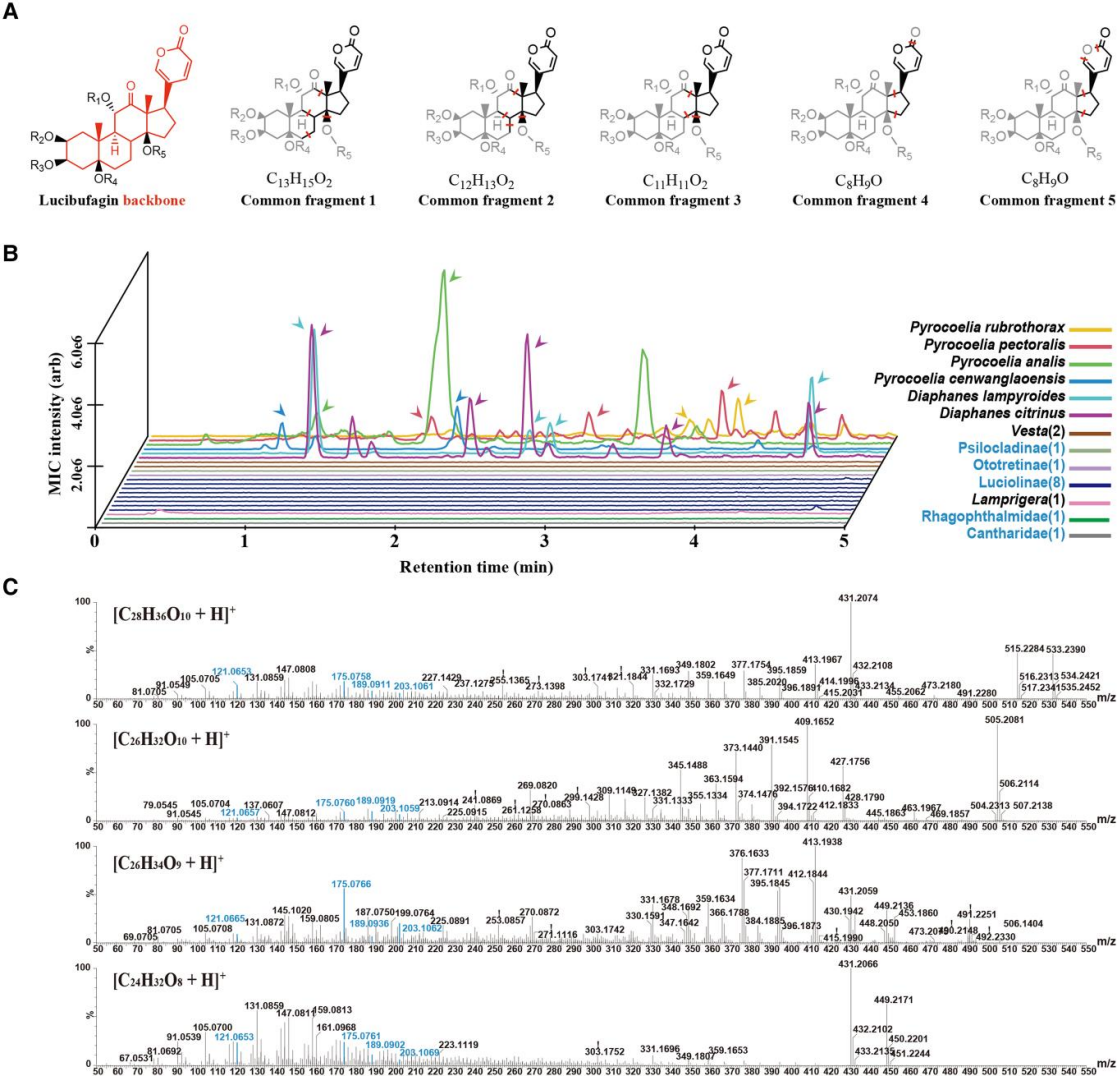
A systematic survey of LBGs across firefly species and outgroups using LC–MS. A) LBG backbone and five LBG common fragments that we identified and used to screen for LBGs. B) LC–HRAM–MS multi-ion chromatograms (MIC) showing the summation of exact parent ion peaks of LBGs of 21 species (tolerance = 5 ppm). The number in parentheses indicates the number of species examined in that group. The arrows highlight confirmed LBG components. C) MSMS spectra of precursor ions of pronated LBGs. Blue highlights peaks of common fragments.

The existence of LBGs in most firefly species is yet to be determined and the biosynthetic pathway of LBGs remains unclear. We first used LC–MS to determine the presence and absence of LBGs in each species through three approaches. In addition to fresh samples of the 18 different species we collected, museum-preserved specimens of 3 relatively rare species in Lampyridae were obtained (Table S1). Untargeted screening was first conducted as there were no LBG standard compounds available. The screening was realized based on MS^E^ data and the self-built LBG library of known LBG molecule structures (Table S4) (25) using UNIFI software. LBGs were found in six species from *Pyrocoelia* and *Diaphanes* (Fig. 2B) and were further confirmed to match theoretical MS spectra (Fig. S2) and tandem mass spectrometry (MSMS) spectra of protonated ions of known LBGs (Fig. 2C) (11, 14). To investigate unknown LBGs that have not been covered by our LBG library, we further performed a common fragment search (46, 47). We first identified five common fragments containing the characteristic six-member lactone ring of LBGs (Fig. 2A). These five common fragments are not affected by variations of the substituent group, so they should be shared by more diverse LBGs (48). The absence of these common fragments in a sample is an indication of the absence of LBGs. We found that except for *Pyrocoelia* and *Diaphanes*, none of the other samples had the co-presence of these five common fragments, confirming the absence of LBGs in these samples (Table S5). In addition, for previously reported LBGs that only had molecular weight or molecular formula available (26, 48), we manually screened the mass spectra by extracting their precise molecular weights in total ion chromatograms based on both MS and MS^E^ mass data. We found multiple mass-spectra-matching molecular weights in samples from *Pyrocoelia* and *Diaphanes* (Table S6), and further used the above common fragment search method to exclude molecules with the same molecular weight but no LBGs' characteristic lactone ring (Table S6).

**Fig. 3.**
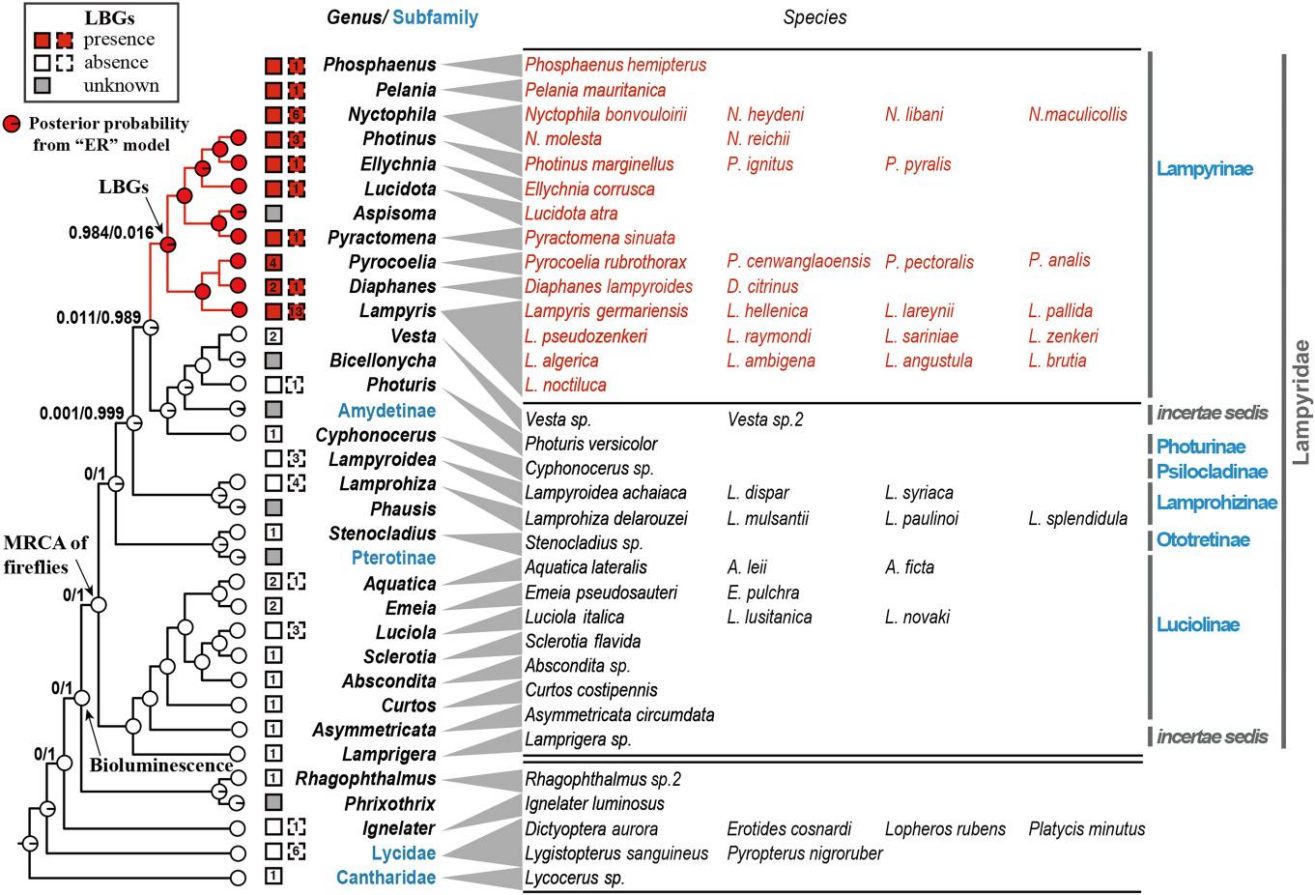
A single origin of LBGs in Lampyrinae revealed by ancestral state reconstructions. The presence of LBGs across firefly species tree. The topology of the tree was compiled from phylogenetic tree in Fig. S1A, Martin et al. (40), Chen et al. (44), and Zhang et al. (49). Each terminal branch leads to a genus (italicized names in black) or (sub)family (names in blue) with either LBGs or genomic information available, or both. Four genera with LBG information but no genomic data were placed into subfamilies without branches leading to them. Inferred ancestral states by ER model are shown by pie charts at the tips and nodes of the tree, with the red and white parts indicating posterior probabilities of the presence or absence of LBGs, respectively. Posterior probabilities are labeled on key ancestral nodes. The number within the squares shows the number of species examined in this study (left) and in previous studies (right). All species examined are listed in the right panel.

In summary, LBGs are found in all six species from genera *Pyrocoelia* and *Diaphanes* in the Lampyrinae subfamily. In contrast, no LBGs were found in any of the 15 representative species in Luciolinae, Ototretinae, Psilocladinae, Lampyridae *incertae sedis* lineages *Vesta* and *Lamprigera*, Rhagophthalmidae, and the outgroup *Lycocerus* (Fig. 2B; Tables S5 and S6). Together with previously reported cases (Fig. 3 and Table S7), we compiled information on the presence and absence of LBGs from a total of 68 species in 25 genera in 6 subfamilies and 2 genera in 2 *incertae sedis* lineages in Lampyridae (Lampyrinae: 11 genera, Luciolinae: 7 genera, Lamprohizinae: 3 genera, Photurinae: 2 genera, Ototretinae: 1 genus, Psilocladinae: 1 genus, *incertae sedis*: *Vesta* and *Lamprigera*), as well as representative species in Rhagophthalmidae, Elateridae, Lycidae, and Cantharidae (Fig. 3). We note that our analysis only considers LBGs that were biosynthesized *de novo*, and not ones that were acquired through sequestration from prey, as in the case of *Photuris* species (33). Together, we found that of the species that have LBG information, LBGs present exclusively in Lampyrinae subfamily (Figs. 2B and 3).

### Origin of LBGs in the common ancestor of Lampyrinae subfamily

There is no detectable level of LBGs in any newly examined or previously reported species from lineages except for Lampyrinae, suggesting that the origin of LBGs may only be traced back to the common ancestor of Lampyrinae (Fig. 3). To test this hypothesis, we reconstructed ancestral states of LBGs across the bioluminescent beetles, including all eight subfamilies of Lampyridae and genera with LBG data, based on phylogenetic relationships from our data and Martin et al. (Fig. 3). Two subfamilies without LBG data were included and marked as unknown LBG status. Genera with LBG data but no phylogenetic information based on genomic data were placed on the phylogeny but not included in the ancestral state reconstructions (Fig. 3). We first estimated the transition matrix between the presence and absence of LBGs under both equal rate (ER) model and all-rates-different (ARD) model using MCMC approach implemented in *phytools* (50). We then calculated the posterior probabilities of the presence or absence of LBGs for internodes using stochastic character mapping method with 2,000 simulations (51). Under ER model, the transition rate between the two states is estimated to be 0.009, and the posterior probabilities of the presence of LBGs in the most recent common ancestor (MRCA) of Lampyrinae subfamily and the MRCA of Lampyridae family are 0.984 and 0.016, respectively (Figs. 3 and S3). Under ARD model, the estimated transition matrix is 0.040 for gain and 0.144 for loss of LBGs. The posterior probabilities of the presence of LBGs in the MRCA of Lampyrinae and the MRCA of Lampyridae are 0.926 and 0.074, respectively (Fig. S3). Both models support with high posterior probabilities that LBGs were present in the common ancestors of Lampyrinae subfamily, but not in the more ancient common ancestors of all fireflies. This result has the important implication that firefly bioluminescence evolved much earlier than firefly toxins and probably did not originally function as a warning signal.

To infer the approximate time of LBG origin, we estimated the divergence times of common ancestor of Lampyrinae and its closest LBG-free sister group Photurinae using MCMCTree from PAML. Three fossil calibration points were used (Table S8), including *Protoluciola albertalleni* at the root of Luciolinae (39), *Cretophengodes azari* at the node Phengodidae + Rhagophthalmidae (52), and *Litholacon*, *Ageratus*, and *Cryptocardius* at the root of Elateridae (53). The divergence times of Lampyrinae and Photurinae were estimated as 93 Mya (95% highest posterior density [HPD]: 81–107 Mya), while the MRCA of Lampyrinae is dated to 66 Mya (95% HPD: 56–76 Mya). The biosynthesis of LBGs should have first evolved after the divergence of Lampyrinae and Photurinae, and before the diversification within Lampyrinae (Fig. 1).

### ATPα from LBG-containing Lampyrinae have higher CTS resistance

**Fig. 4.**
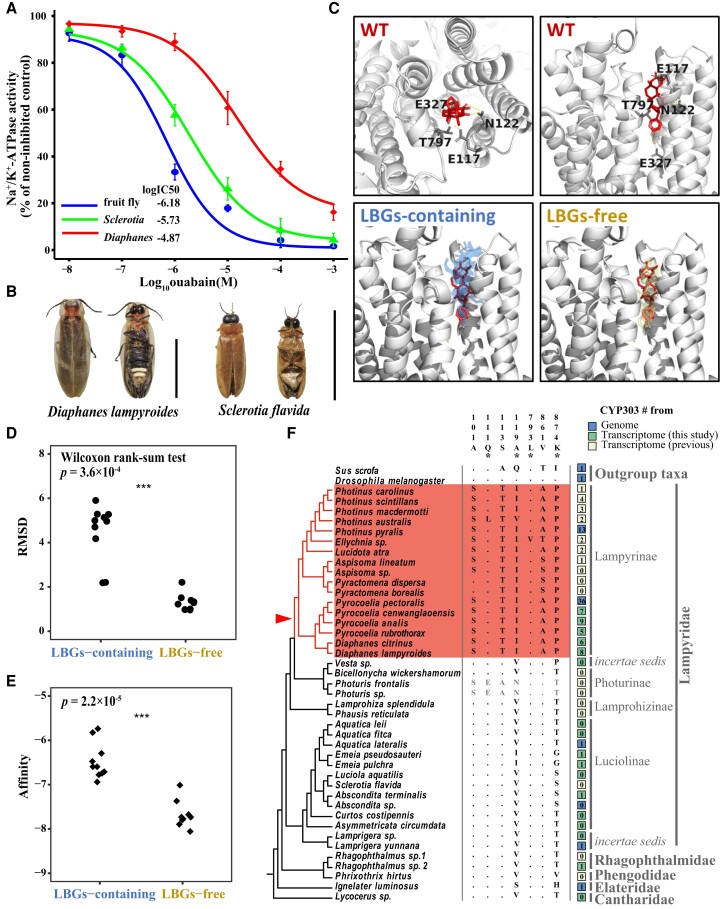
Amino acid substitutions in ATPα of LBG-containing Lampyrinae fireflies that may reduce its sensitivity to LBGs. A) NKA in vitro inhibition assays. The data points represent the mean of three biological replicates (±SE). B) Adult fireflies of *D. lampyroides* and *S. flavida.* Scale bar: 1 mm. C) Top row: pig ATP1A1 bound to core LBG (in red). Bottom row: molecular docking of core LBG onto predicted structures of native ATPα from LBG-containing firefly species (in light blue) and LBG-free firefly species (in light yellow). The best docking positions for each native ATPα in Table S10. The core LBG-ATP1A1 position in red is displayed as reference. The yellow dotted lines show possible hydrogen bonds between ATP1A1 and core LBG. All ligand–protein interactions formed on the β-surface of core LBG and bufalin are listed in Table S9. D) The RMSD and E) docking scores (affinity) of core LBG are significantly different between native ATPα of LBG-containing and LBG-free fireflies. F) Substitutions in ATPα in LBG-containing Lampyrinae. The numbers in the top panel correspond to the position in mature pig enzyme ATP1A1. Only sites with substitutions previously implicated in CTS tolerance (*), or that are Lampyrinae specific and Lampyrinae prevalent, are displayed. The dots represent identity with the consensus sequence. The red shade LBG-containing Lampyrinae species and the red triangle indicate the faster evolutionary rate of ATPα in the LBG-containing Lampyrinae. The colored squares on the right denote the number of CYP303 genes that we identified from genomes and transcriptomes.

CTSs, including LBG toxins, directly bind to and inhibit ATPα that have essential and conserved functions across animals. Parallel evolution of ATPα target-site insensitivity has been reported in multiple CTS-resistant insects (54–56). Recent study has also reported that ATPα target-site insensitivity plays important roles in CTS resistance in *Photuris* and *Photinus* fireflies (57). ATPα across Lampyridae is highly conserved, with at least 91% amino acid identity. We estimated that *d*_N_/*d*_S_ is 0.023 for the branch leading to LBG-containing Lampyrinae, which is much higher than the background *d*_N_/*d*_S_ ratio of 0.009 across the rest of the phylogeny. The two-ratio model fits significantly better to our data than the one-ratio model using PAML (*P* = 2.75 × 10^−120^), suggesting that ATPα evolved at a faster rate in the LBG-containing Lampyrinae and may be under positive selection (Fig. 4F). Using branch-site model and site model, we did not identify any specific site under positive selection in species with LBGs.

To examine whether LBG-containing Lampyrinae have ATPα that is more CTS-resistant compared with LBG-free firefly species, we performed in vitro NKA activity assays under different concentrations of CTS ouabain, using representative species *Diaphanes lampyroides* and *Sclerotia flavida* (Fig. 4A and B). The in vitro assays confirmed that ATPα from LBG-containing *D. lampyroides* is more resistant to ouabain (7.2-fold), compared with ATPα from LBG-free *S. flavida* (Fig. 4A).

We then used molecular docking to examine the interaction of LBG toxin with ATPα from LBG-containing and LBG-free fireflies. We first simulate crystal structure of core LBG and pig ATP1A1 complex using highest resolution pig ATP1A1–bufalin crystal complex (PDB accession: 4RES) as template. The best docking structure of core LBG into native CTS-sensitive pig ATP1A1 shares high similarity with pig ATP1A1–bufalin crystal complex in both docking position and docking pocket (Fig. S4). The β-surface of core LBG interacts with residues E117, N122, E327, and T797 (Fig. 4C and Table S9). We next performed docking simulations of core LBG onto several native ATPα from both LBG-containing Lampyrinae fireflies and LBG-free fireflies. We found that the best docking positions of core LBG molecules shift outward from the binding pocket on ATPα from LBG-containing fireflies, compared with that from LBG-free fireflies (Fig. 4C and D). Additionally, ATPα in LBG-containing Lampyrinae fireflies has significantly lower affinity to core LBG than ATPα in LBG-free fireflies (Wilcoxon test, *P* = 2.2 × 10^−5^; Fig. 4E and Table S10).

In LBG-containing Lampyrinae species, for sites previously implicated in CTS sensitivity of ATPα, amino acid substitution are only found at four sites, i.e. Q111L, V119I, L793V, and T874P (Fig. 4F) (54, 55, 58). These substitutions were reported to modestly increase CTS resistance, and two of them present in only one species, i.e. Q111L in *Photinus australis* and L793V in *Ellychnia* sp. Additionally, for the rest of sites previously unknown to affect CTS resistance, we searched for candidate substitutions that co-evolved with LBGs in the common ancestor of Lampyrinae that may confer CTS resistance, specifically, substitutions present in at least 15 of the 17 Lampyrinae species surveyed, but absent in the LBG-free species, the sensitive pig ATP1A1, and *Drosophila melanogaster* ATPα. Using these criteria, in addition to V119I and T874P, we identified substitutions A101S, S113T, and V861A/S that are specific and prevalent in LBG-containing Lampyrinae fireflies (Fig. 4F). *Photuris* species evolved LBG resistance independently under distinct ecological contexts and were thoroughly investigated in a separate study (57).

We used molecular docking to explore the effects of the five Lampyrinae-prevalent candidate substitutions in ATP1A1 on its LBG sensitivity, i.e. A101S, S113T, V119I, V861A, and T874P. We introduced each of the five candidate substitutions to pig ATP1A1, and performed docking simulations of core LBG onto pig ATP1A1 carrying a specific candidate substitution. We found that all five substitutions could result in a reduction in binding affinity compared with the wild-type docking confirmation (Fig. S5 and Table S11). Additionally, substitution A101S allows core LBG to penetrate deeper into the binding pocket, likely destroying the hydrogen bond between core LBG and N122. Substitution T861A may influence LBG and ATP1A1 interaction by rotating the core LBG and forming new hydrogen bonds with A323, which may lead to the decreased binding affinity. These results, together with the in vitro enzyme assays, suggested that ATPα from LBG-containing fireflies is less sensitive to LBG and target-site insensitivity may contribute to LBG-containing fireflies to deal with LBG toxins in their bodies.

## Discussion

To shed light on the hypothesis that firefly bioluminescence originally evolved as warning signals to their toxic LBGs, we examined the evolution of LBGs by systematically surveying LBGs and reconstructing confident phylogenetic relationships in fireflies and outgroups. We report for the first time that LBGs are present in five species from two genera in Lampyrinae subfamily, but more notably, absence in all other surveyed lineages, including eight species from six genera in Luciolinae subfamily, species in subfamilies of Ototretinae and Psilocladinae and two Lampyridae *incertae sedis* lineages of *Lamprigera* and *Vesta*, as well as outgroup Rhagophthalmidae family. Combined with published data, we find that LBGs are present in all examined species in Lampyrinae subfamily, while none of the species from other firefly lineages have detectable levels of LBGs. Ancestral state reconstructions based on a confident species tree constructed using genomic-level data support the high posterior probabilities that LBGs evolved once within fireflies in the common ancestor of Lampyrinae, much later than the origin of bioluminescence in the common ancestors of all fireflies. Therefore, bioluminescence did not originate as a warning signal to LBG toxins. Furthermore, we combined in vitro NKA enzyme activity assays and molecular docking simulations to demonstrate that ATPα in LBG-containing species is more resistant to CTS, which may contribute to the ability of these species to tolerate the high levels of LBGs in their bodies.

Currently, there is little evidence to support the hypothesis that bioluminescence originally evolved in the common ancestors of fireflies as a warning signal. It is particularly challenging to infer the ancestral function of a novel trait when it first evolved, as it may differ from the present primary function of the focal trait. However, an intriguing study has suggested that glowing millipedes may have initially developed bioluminescence to cope with oxidative stress in hot, dry environment, and was later co-opted as aposematic signals (5). Additionally, previous studies have also found that firefly luciferin could act as an antioxidant against oxidative stress (59, 60). The MRCA of fireflies dates back to 136 Mya (95% HPD: 122–153 Mya) and the MRCA of Lampyridae, Rhagophthalmidae and Phengodidae dates back to 183 Mya (95% HPD: 161–208 Mya) (Fig. 1). During this time period, the oxygen level in the atmosphere continued to rise from a historical low level at ∼190 Mya (Fig. 1) (43, 61). The continental environment was inferred to be hot with extreme wet/dry seasons after Toarcian Oceanic Anoxic Event at ∼182 Mya (62). Based on this information, we hypothesize that luciferin and the bioluminescence reaction might have originally evolved to cope with the increasing oxidative stress and the hot, dry environment, and were later adapted to their current biological functions, such as warning and mating signals. A recent study reported multiple parallel origins of bioluminescence in beetles (10, 11). Future investigation and additional evidence are needed to test this hypothesis and determine whether it is applicable to multiple origins across lineages.

In addition to target-site insensitivity of ATPα in LBG-containing species, additional mechanisms may also contribute to the ability of these species to tolerate the high levels of LBGs in their bodies. Firstly, there may be physical isolation of LBGs and ATPα. The LBG toxins are present in high concentrations in the hemolymph (11), which circulates throughout the whole body and bathes all the internal organs in the open circulatory system. Physical barriers, such as glial sheath surrounding nervous tissue or the blood–brain barrier, may keep LBG toxins away from ventral nerve cord or the brain, which have a high expression of ATPα (63). This would be consistent with previous finding that several herbivores feeding on and sequestrating cardenolides from milkweeds also have relative sensitive ATPα expressed in the brain, compared with the gut and muscle (54, 58). Secondly, active efflux transporters could remove CTSs from sensitive tissues, such as nerves (64) or malpighian tubes (65). Moreover, several classes of enzymes are also known to play important roles in general detoxification (66).

LBGs may exhibit greater diversity than currently known. The untargeted screening based on custom-built LBG library with known structures is effective in identifying specific LBG toxins, but unknown LBGs may diverge sufficiently from known varieties to evade detection using this approach. To address this limitation, we have implemented a common fragment search targeting the characteristic six-member lactone ring in backbone structure of LBGs. Specifically, five common fragments were used, which should all be present when LBGs with the lactone ring are present and are unaffected by changes in the substituent group. Additionally, we have screened for precise molecular weights of all reported LBGs. However, for most of these reported compounds, the lack of molecular structures and MSMS profiles from standards makes it difficult to establish conclusive matches based on molecular weights alone. Thus, the common fragment method was used to further exclude matches that are not LBGs. Lastly, while the quantity and profile of LBGs in each LBG-containing species appear to be variable (Fig. 2A), but the unavailability of LBG standards precludes the determination of absolute quantities. The observed differences in LBG profile suggest there might be different LBG modification enzymes in different species, or it could be due to individual differences.

The biosynthesis pathway of LBGs in fireflies remains unclear. Recently, through a comparison of the sequenced genomes of LBG-containing *P. pyralis* and LBG-free *A. lateralis*, a CYP303 gene family was found to expand drastically in LBG-containing *P. pyralis* (11). This CYP303 gene family, which belongs to the P450 family, was suggested to potentially participate in the oxidation reaction in the biosynthesis of LBGs, although this has not yet been tested by functional experiments. From whole-body transcriptomes we collected for multiple LBG-containing Lampyrinae and LBG-free species, we found at least five copies of this CYP303 ortholog in LBG-containing species, and no more than one single copy from LBG-free species (Figs. 4F and S6), suggesting that CYP303 gene family expansion might co-evolve with LBGs at the common ancestors of LBG-containing Lampyrinae. However, due to the temporal and tissue-specific nature of transcriptomes, we refrain from comparing our data from the whole body to the low number of CYP303 orthologs obtained from primarily tissue-specific datasets curated from other published studies. It is important to note that the evolution of CYP303 gene family is still inconclusive and warrants future investigation utilizing whole genome data, considering the incomplete nature of transcriptome data.

LBGs are well-known and the most studied firefly toxins reported in multiple species. As far as we know, no other toxin has been reported in more than a single firefly species; however, we could not completely rule out the possibility that bioluminescence might originally evolve as warning signals for other common firefly toxins or ancestral forms of toxins. A couple of potentially toxic compounds has been reported in individual firefly species. For example, *Photuris* have been found to contain a potentially toxic substance betaine *N*-methylquinolinium 2-carboxylate (27, 67). Additionally, larvae of *Luciola* have been reported to release resin- or peppermint-scented secretions from eversible segmental sacs (68). Two volatile terpenoids, i.e. terpinolene and γ-terpinene, were identified in the glandular secretion from larvae of aquatic firefly *Aquatica leii*, and might serve as deterrents against fish and ants (69). *N*-methylquinolinium 2-carboxylate was not found at detectable levels in any of the species we examined using LC–MS, including *Vesta* sp., the closest sister species of *Photuris* group. This suggests that *N*-methylquinolinium 2-carboxylate may be unique to previously examined *Photuris* species. In a preliminary screening using GC–MS method, we only found terpinolene in larvae of *A. leii*, but not in larvae of *Pyrocoelia analis*, *S. flavida*, *Pygoluciola* sp., and adult male of *A. leii* (Fig. S7). These findings suggest that the two previously reported potential toxins are likely even more restricted to specific firefly species or genus.

Due to the mysterious lifestyles of immature stages of most firefly species, the nonmobile or less-mobile stages of most fireflies, including pupae, larvae, and adult females of some species, are very difficult to find and collect in the field. For this reason, previous studies (Table S7) and our study examined LBG toxins primarily in adult male samples. For a few studies that used samples other than adult males (Table S7), LBGs are found to be present at all life stages and in both sexes in the winter firefly (25), and in larvae and adult males of *P. pyralis* (11). However, the relative amount of LBGs across stages and sexes was not compared. It has also been reported that *Photinus* nuptial gifts contain LBGs (70). We had the chance to examine a very limited number of samples from the immature stages of *Py. analis*, and we preliminarily found that eggs, larvae, and pupae all contain LBGs, and the amount per individual seems to be relatively higher in adult males. Future investigations of stage and sex-specific LBGs would help improve our understanding of the ecological role and dynamics of the biosynthesis of LBGs in fireflies.

## Materials and methods

### Sampling and species identification

Adult samples of 18 fresh species were collected from diverse locations across China, including 16 species from Lampyridae family, 1 species from Rhagophthalmidae family, and 1 outgroup species from Cantharidae family (Table S1). No permits were required at the time of collection, and no endangered or protected species were involved. Samples were first identified as species by morphological characteristics, flashing patterns, and flight behavior (71, 72). When available, adult male genitalia and aedeagal sheath were dissected and examined under a microscope (SZ650 microscope Optec). Species identities were further verified using CO1 barcoding sequences. CO1 sequences for each species were assembled from RNA-seq data using Mitofinder (v1.4) with default parameters. The assembled DNA sequence from each sample was searched against BOLD database and NCBI nr database for matched species with >95% identity (73–75). Three new species were identified and reported (41, 42). In addition, we obtained three museum-preserved specimens, one each from subfamily Ototretinae, Psilocladiane, and genus *Vesta*, from the Museum of Biology, Sun Yat-sen University (Table S1).

### Transcriptome sequencing and assembly

Fresh samples were stored in RNAlater (Qiagen, Valencia, CA, USA) at −80 °C. For most species, an adult male sample was used because female samples of many species were more difficult to collect in the field, except *Rhagophthalmus* sp. 2 and *Lamprigera* sp. for which only adult female and larva sample were available (Table S1). Total RNA was extracted for each of the 16 species using Eastep Super Total RNA Extraction Kit (Promega, Shanghai) following manufacturer’s protocols. RNA-seq libraries were prepared and indexed using NEBNext Ultra RNA Library Prep Kit for Illumina (NEB, USA). All libraries were sequenced with paired-end 150 bp reads on the Illumina NovaSeq 6000 platform (Novogene Bioinformatics Institute, Beijing, China).

We used FASTQC v0.11.8 (76) to assess the quality of RNA-seq raw reads. Transcriptome for each species was de novo assembled using Trinity (v2.8.4) with default parameters and option –trimmomatic (77, 78). Coding regions were predicted using TransDecoder (v5.0.2; default parameters, https://transdecoder.github.io) implemented in Trinity.

### LBG identification by LC–MS

For each of the 18 collected species, LBGs were examined in alive adult males of 16 species, adult females for *Rhagophthalmus* sp. 2, and larva for *Lamprigera* sp. Each sample was homogenized with a micropestle in a 1.5-mL microcentrifuge tube in 500 μL 100% acetonitrile, and then centrifuged at 13,523×*g* for 5 min. The supernatant was collected and filtered through a 0.22-μm PFTE filter (no. 721-1320, Thermo Scientific Nalgene) and stored at −20 °C. For three dry firefly specimens, LBGs were extracted as described in Berger et al. (26).

The presence of LBGs in each filtered supernatant was examined on an ultra-performance LC–time-of-flight mass spectrometer (I-class Synapt XS, Waters, USA) using an Acquity UPLC T3 column (1.8 μm × 2.1 mm × 50 mm). The mobile phases were 0.1% formic acid in H_2_O (solvent A) and 0.1% formic acid in acetonitrile (solvent B) with the gradient elution consisting of initial 15% solvent B, increasing to 25% solvent B within 3 min, increasing to 80% solvent B within 3 min, holding for 2 min, switching back to initial 15% solvent B and holding for 2 min. The flow rate was maintained at 0.4 mL/min, and the column temperature was 35 °C. For MS, all samples were analyzed in positive ion mode using an electro-spray ionization source. A high voltage of 2.5 kV was applied for ionization, and the voltages applied to sampling cone and source offset were 20 and 60 V, respectively. The source temperature and desolvation temperature were 150 and 400 °C, respectively. High nitrogen flow of 900 L/h was used for desolvation. MS, MS^E^, and MSMS mass data were acquired by MassLynx (v 4.2) with *m*/*z* ranging from 50 to 1,000 Da. MS^E^ functional data were used in untargeted screening search. MS^E^ function contains low- and high-energy channels. No collision energy was applied at the collision trap in the low-energy channel, while collision energy ramped from 60 to 80 V was applied in the high-energy channel.

For known LBGs, an LBG library based on eight previously reported LBG structures was created using UNIFI software (v1.9.4.053; Table S4) (25). For data screening of injected samples, the mass tolerance was set to ±5 ppm, and the adduct type is +H or +Na. For every identified component in the component plot, parent and fragment ions were atomically assigned in low- and high-energy mass spectra (Figs. S8 and S9). To further determine the identified LBGs screened via UNIFI, MSMS analysis was performed. The identified candidates for LBG compounds were further confirmed by theoretical MS spectra and MSMS analysis.

Common fragment search was performed to examine more diverse unreported LBGs (46, 47). The core molecular structure of the LBG class of compounds has been proposed (Fig. 2A) (48). Lucibufagin C ([M + H]^+^ = C_28_H_37_O_10_, *m*/*z* = 533.2386) was reported in several Lampyrinae fireflies, and its MSMS spectrum has been most extensively studied (14). The MSMS fragment peak lists of LBGs with Lucibufagin C as an example were analyzed, using the mass fragment function implemented in MassLynx (v 4.2). We identified five mass fragments related to LBG backbone from the characteristic six-member lactone ring that are not affected by changes in the substituent group (Fig. 2A). Their co-presence was used to screen for the presence of LBGs in our data using the UNIFI software (v1.9.4.053) (11, 79). In addition, for previously reported LBGs with only molecular formula or molecular weight (26, 48), theoretical molecular weight was calculated based on molecular weight, and samples were manually screened (*m*/*z* tolerance = 1 mDa) for masses calculated LBGs in MassLynx (v 4.2).

### Phylogenomic analysis

The longest isoform per gene from de novo assembled transcriptomes was extracted using custom script. For species with genomes available (Table S2), protein sequences were downloaded and CD-HIT (-c 0.9 -n 5) was used to remove redundancy (80, 81). OrthoFinder (v2.2.6) was then used to identify orthologs among all 41 species with default parameters (Lampyridae: 36; Rhagophthalmidae: 2; Phengodidae: 1; Elateridae: 1; Cantharidae: 1) (82). We identified single-copy orthologs, allowing 20% missingness across all species.

For each orthogroup, DNA-coding sequences were aligned using MAFFT (v7.480) (83) in TranslatorX (84). Poorly aligned sites were filtered by GBLOCKS (0.91b) using (-t=p -b4=10 -b5=n) (85, 86). Before phylogeny reconstruction, we used PartitionFinder2 (87) to conduct partition analysis and estimate the best substitution model for each partition. All alignments were concatenated to a phylip-formatted supermatrice as an input file, and then, partition was conducted based on data blocks defined by the sites of each gene. Different partitioning schemes were tested based on these data blocks and sets of sites that might have evolved in a similar way were found (87). The optimal partitioning scheme and corresponding substitution model for each partition were selected by Bayesian information criterion under the “rcluster” search algorithm (88) with equal weighting for overall rates, base frequencies, model parameters, and the alpha parameter. PartitionFinder yielded the best partitioning scheme with 85 subsets (80 GTR + G + I and 5 GTR + G nucleotide substitution models) for the concatenated DNA matrix or 161 subsets (50 LG4X, 37 LG + X, 28 LG + I + G, 27 LG + G, 9 LG + I + G + X, 7 LG4M + G, and 3 LG amino acids substitution models) for the concatenated amino acid matrix. Using the best partitioning scheme suggested by PartitionFinder, we inferred the nucleotide tree using the GTRGAMMAI model for each partition and estimated the node support values by 1,000 rapid bootstraps in RAxML 8.2.4 (89). Similarly, we infer the amino acid phylogeny using the best partitioning scheme and the PROTGAMMALGX model for each partition with 100 rapid bootstrap replicates. The phylogenetic tree was displayed in FigTree (v1.4.4) (http://tree.bio.ed.ac.uk/software/figtree/).

### Divergence time estimation

Three documented fossils were used to calibrate divergence times (Table S8). (i) *Litholacon*, *Ageratus*, and *Cryptocardius* (53, 90): Fossils of these genera could be dated back to the upper Jurassic of Karabas (166.1–157.3 Mya) and were placed in subfamily Agrypninae of Elateridae. Thus, we constrained the minimum stem age of Agrypninae (split of Elateridae + Phengodidae) at 157.3 Mya. (ii) *C. azari* (52): It was placed in a new bioluminescent Elateroid beetle family Cretophengodidae from the Middle Cretaceous of northern Myanmar (99 Mya) which is sister to the Phengodidae + Rhagophthalmidae clade. This fossil could be used to calibrate the minimum age of the split of the Phengodidae and Rhagophthalmidae. (iii) *Pr. albertalleni* (39): A fossil firefly was placed to the Luciolinae and was described from a Cretaceous Burmite amber (100 Mya). We placed it at the stem of Luciolinae (split of Luciolinae + *Lamprigera*) to calibrate the minimum age of this clade in our phylogeny.

We first estimated a mean substitution rate for the all sequences using BASEML from PAML with a strict molecular clock and the ML tree derived from RAxML 8.0 and constraint of 200 Mya to root based on our preliminary calibration analyses. The estimated substitution rate (0.0024 substitution/site/Mya) was used to inform the “regene_ gamma” prior in MCMCTree. We then estimated divergence times using approximate likelihood method in MCMCTree following the two-step procedure based on topology of ML tree, concatenated alignments, and fossil calibrations (91). We concatenated alignments that have three partitions (first, second, and third codons, ndata = 3) and assigned an HKY85 + G model (model = 4) to each partition with five gamma categories (ncatG = 5) and the following parameter values: alpha = 0.1, BDparas = 1 1 0, kappa_gamma = 6 2, alpha_gamma = 1 1, regene_gamma = 2 8.27 1, sigma2_gamma = 2 5 1, and finetune = 1. The time unit used in this analysis is 100 million years. Fossil calibrations were assigned at the nodes of phylogeny under a truncated Cauchy distribution with parameters as follow: *t_L_* = fossil ages, *P* = 0.1, *c* = 0.2, *p_L_* = 0.025. In addition, we specified a loose constraint on the age of the root with 250 Mya (the early Jurassic) as maximum bound and 157.3 Mya (the age of Litholacon fossils) as a minimum bound because no such fossil constraint is available. We performed 2 separate analyses with different random seeds with a burning of 250,000 iterations and sampling every 250 iterations. Finally, we collected 80,000 samples from each run and then assessed convergence using r script from github.com/mariodosreis/divtime. The effective sample size for all parameters in each run was ≥1,000.

### Ancestral state reconstruction

Stochastic character mapping (51) was used to estimate posterior probabilities of ancestral states for the presence and absence of LBGs along a phylogeny integrated from our data and Martin et al. We determined tip states based on publications and our LC–MS results. Tip states were assigned using a matrix with prior probabilities for character state in rows and character states as column names. For tip without LBG data, we marked it as an unknown state by an equal probability of the presence or absence of LBGs (0.5, 0.5). We first estimated the transition matrix between the presence and absence of LBGs under both ER model and ARD model, as well as priors on the root of phylogeny, using the MCMC with 100,000 iterations and sampling every 50 iterations after the first 10,000 iterations burn-in. Stochastic character maps were simulated over the posterior distribution of the transition matrix and thus account for uncertainty in transition rates. We then estimated the posterior probabilities of states for internodes and tips with unknown status based on these MCMC samplings. Stochastic character maps were simulated using *make.simmap* function and summarized using describe.simmap function in *phytools* package (50).

### Identification of ATPα homologs

Homologous ATPα transcripts for each species were identified using *P. pyralis* ATPα protein sequence (GenBank: XP_031345330) as a query to search against the *de novo* assembled transcriptomes using TBLASTN (cutoff *e*-value: 1e−10) (92). The longest isoform per gene was chosen when applicable. We then mapped all raw reads to the longest isoform and manually examined the accuracy of the assembly and extended the sequences when possible in GENEIOUS (v9.0.5) (93). Finally, each identified candidate ATPα sequence was BLASTed against the NCBI nr database with default parameters, and only sequences with the best match to annotated ATPα homologs were retained. ATPα protein sequences for pig (*Sus scrofa*, NP_999414.1) and fruit fly (*Dr. melanogaster,* AAC05260.1) were downloaded directly from NCBI. Translated coding sequences were aligned using MUSCLE algorithm (94) in GENEIOUS. The quality of alignment was manually examined and corrected when needed.

### Molecular evolution of ATPα using PAML

Positive selection can be detected by assessing the ratio of nonsynonymous substitutions to synonymous substitutions (*d*_N_/*d*_S_). A lineage that underwent positive selection may have a *d*_N_/*d*_S_ that is higher from background lineages. A two-ratio model in codeml of PAML was used to detect a significant change in the evolutionary rate of ATPα in LBG-containing fireflies compared with background lineages (95). The internal branch leading to Lampyrinae clade is set as the foreground branch. The one-ratio model M0 was set as the null hypothesis. To access whether the two-ratio model fits our data significantly better, the *P*-values were estimated assuming a null distribution that is a 50:50 mixture of a χ^2^ distribution and a point mass at zero.

### Molecular docking simulations

Molecular docking simulations were used to examine binding affinity and interactions between core LBG and native ATPα from LBG-containing and LBG-free fireflies, as well as ATP1A1 carrying specific substitutions. The crystal structure of bufalin-bound pig ATP1A1 (PDB:4RES, 3.41 Å) is the highest resolution CTS bufadienolides bound ATP1A1 structure available (96) and was used as our template (Fig. S4). Water molecules and bufalin were first extracted from the crystal structure. To perform molecular docking, the structure of the core LBG ligand was generated with explicit polar hydrogens and optimized with energy minimization in MOE package (97). Homology modeling for native firefly ATPα and ATP1A1 carrying specific substitutions was performed with Modeller v9.10 (98) using 4RES as a template. Best models were selected based on quality using Swiss-Pdb Viewer (http://www.expasy.org/spdbv/). For native firefly ATPα from various firefly species, we also performed the structure prediction with AlphaFold2 (99). The ATPα proteins alignment results indicate that the RMSDs of the predicted structures from AlphaFold2 and Modeller in the transmembrane domain are <1 Å (Fig. S10). We thus only used structures from Modeller for following docking simulation. Rigid docking of core LBG and ATP1A1 homologs was performed using the MOE package. In flexible molecular docking, 100 ligand conformations were generated using the “London dG” scoring function, then submitted to a refinement step-based molecular mechanics and rescored with the “GBVI/WSA dG” scoring function. The top docking poses were selected based on docking scores and RMSDs. Docking scores for core LBG binding to ATP1A1 homologs are used to assess the binding affinity. The RMSDs were calculated using the LBG-bound ATP1A1 complex as the reference structure. The interaction between core LBG and ATP1A1 homologs was analyzed and visualized using PyMOL (100).

### In vitro assay of NKA

NKA activity was determined by photometrically measuring the inorganic phosphate released from enzymatically hydrolyzed ATP under increasing concentrations of CTS ouabain. Sample preparation and enzyme inhibition assays were performed, as described previously with modifications (101). Briefly, 10–15 firefly brains were freshly dissected and homogenized in 400 μL precooled deionized water. Undissolved residues were removed by centrifugation at 5,000*×g* at 4 °C for 10 min, and the supernatant was split into three technical replicates. Ouabain-sensitive *Dr. melanogaster* (50 pooled heads for 1 assay) were included as a control. Samples were incubated at concentrations of 10^−8^–10^−3^ M ouabain solutions (with 100 mM NaCl, 20 mM KCl, 4 mM MgCl_2_, 50 mM imidazole, and 2.5 mM ATP) at 37 °C for 20 min. A noninhibited positive control without the addition of ouabain and a negative control without KCl to correct for background were also set up. Each biological replicate was averaged over three technical replicates, and three biological replicates were measured per species. Relatively, activities of NKA were estimated as (abs[full activity] − abs[inhibited activity])/(abs[full activity] − abs[background activity]) and plotted with log_10_(ouabain concentration) in R software, as described by Wang et al. (56).

## Supplementary Material

pgae215_Supplementary_Data

## Data Availability

Raw sequence reads are deposited in the NCBI SRA database (BioProject PRJNA906471).

## References

[1] Lau ES, Oakley TH. 2021. Multi-level convergence of complex traits and the evolution of bioluminescence. Biol Rev. 96:673–691. 33306257 10.1111/brv.12672

[2] Haddock SH, Moline MA, Case JF. 2010. Bioluminescence in the sea. Annu Rev Mar Sci. 2:443–493. 10.1146/annurev-marine-120308-081028 21141672

[3] De Cock R, Matthysen E. 1999. Aposematism and bioluminescence: experimental evidence from glow-worm larvae (Coleoptera: Lampyridae). Evol Ecol. 13:619–639.

[4] Underwood TJ, Tallamy DW, Pesek JD. 1997. Bioluminescence in firefly larvae: a test of the aposematic display hypothesis (Coleoptera: Lampyridae). J Insect Behav. 10:365–370.

[5] Marek PE, Moore W. 2015. Discovery of a glowing millipede in California and the gradual evolution of bioluminescence in Diplopoda. Proc Natl Acad Sci U S A. 112:6419–6424. 25941389 10.1073/pnas.1500014112 PMC4443369

[6] Mcdermott FA . 1917. Observations on the light-emission of American lampyridæ: the photogenic function as a mating adaptation; 5th paper. Can Entomol. 49:53–61.

[7] Zarubin M, Belkin S, Ionescu M, Genin A. 2012. Bacterial bioluminescence as a lure for marine zooplankton and fish. Proc Natl Acad Sci U S A. 109:853–857. 22203999 10.1073/pnas.1116683109 PMC3271926

[8] Lewis SM, Faust L, De Cock R. 2012. The dark side of the light show: predators of fireflies in the Great Smoky Mountains. Psyche (Stuttg). 2012:1–7.

[9] Viviani VR . 2002. The origin, diversity, and structure function relationships of insect luciferases. Cell Mol Life Sci. 59:1833–1850. 12530517 10.1007/PL00012509 PMC11337554

[10] He J, et al 2024. Multiple origins of bioluminescence in beetles and evolution of luciferase function. Mol Biol Evol. 41:msad287. 38174583 10.1093/molbev/msad287 PMC10798137

[11] Fallon TR, et al 2018. Firefly genomes illuminate parallel origins of bioluminescence in beetles. Elife. 7:e36495. 30324905 10.7554/eLife.36495 PMC6191289

[12] Branham M . 2003. The origin of photic behavior and the evolution of sexual communication in fireflies (Coleoptera: Lampyridae). Cladistics. 19:1–22. 34905865 10.1111/j.1096-0031.2003.tb00404.x

[13] Sivinski J . 1981. The nature and possible functions of luminescence in Coleoptera larvae. Coleopt Bull. 35:167–179.

[14] Tyler J, Mckinnon W, Lord GA, Hilton PJ. 2008. A defensive steroidal pyrone in the glow-worm *Lampyris noctiluca* L. (Coleoptera: Lampyridae). Physiol Entomol. 33:167–170.

[15] Bessho-Uehara M, Konishi K, Oba Y. 2017. Biochemical characteristics and gene expression profiles of two paralogous luciferases from the Japanese firefly *Pyrocoelia atripennis* (Coleoptera, Lampyridae, Lampyrinae): insight into the evolution of firefly luciferase genes. Photochem Photobiol Sci. 16:1301–1310. 28660982 10.1039/c7pp00110j

[16] Long SM, et al 2012. Firefly flashing and jumping spider predation. Anim Behav. 83:81–86.

[17] Leavell BC, et al 2018. Fireflies thwart bat attack with multisensory warnings. Sci Adv. 4:eaat6601. 30140743 10.1126/sciadv.aat6601 PMC6105302

[18] De Cock R, Matthysen E. 2003. Glow-worm larvae bioluminescence (Coleoptera: Lampyridae) operates as an aposematic signal upon toads (*Bufo bufo*). Behav Ecol. 14:103–108.

[19] Jones FM . 1932. Insect coloration and the relative acceptability of insects to birds. Trans R Entomol Soc Lond. 80:345–371.

[20] Lloyd JE . 1973. Firefly parasites and predators. Coleopt Bull. 27:91–106.

[21] Blum MS, Sannasi A. 1974. Reflex bleeding in the lampyrid *Photinus pyralis*: defensive function. J Insect Physiol. 20:451–460.

[22] Eisner T, Wiemer DF, Haynes LW, Meinwald J. 1978. Lucibufagins: defensive steroids from the fireflies *Photinus ignitus* and *P. marginellus* (Coleoptera: Lampyridae). Proc Natl Acad Sci U S A. 75:905–908. 16592501 10.1073/pnas.75.2.905 PMC411366

[23] Eisner T, Goetz MA, Hill DE, Smedley SR, Meinwald J. 1997. Firefly “femmes fatales” acquire defensive steroids (lucibufagins) from their firefly prey. Proc Natl Acad Sci U S A. 94:9723–9728. 9275191 10.1073/pnas.94.18.9723 PMC23257

[24] Gronquist M, et al 2006. Shunning the night to elude the hunter: diurnal fireflies and the “femmes fatales”. Chemoecology. 16:39–43.

[25] Smedley SR, et al 2017. Bufadienolides (lucibufagins) from an ecologically aberrant firefly (*Ellychnia corrusca*). Chemoecology. 27:141–153.

[26] Berger A, Petschenka G, Degenkolb T, Geisthardt M, Vilcinskas A. 2021. Insect collections as an untapped source of bioactive compounds—fireflies (Coleoptera: Lampyridae) and cardiotonic steroids as a proof of concept. Insects. 12:689. 34442254 10.3390/insects12080689 PMC8396437

[27] González A, Schroeder F, Meinwald J, Eisner T. 1999. N-methylquinolinium 2-carboxylate, a defensive betaine from *Photuris versicolor* fireflies. J Nat Prod. 62:378–380. 10075794 10.1021/np980400o

[28] Fu X, Meyer-Rochow VB, Tyler J, Suzuki H, De Cock R. 2009. Structure and function of the eversible organs of several genera of larval firefly (Coleoptera: Lampyridae). Chemoecology. 19:155–168.

[29] Gao H, Popescu R, Kopp B, Wang Z. 2011. Bufadienolides and their antitumor activity. Nat Prod Rep. 28:953–969. 21416078 10.1039/c0np00032a

[30] Schoner W . 2002. Endogenous cardiac glycosides, a new class of steroid hormones. Eur J Biochem. 269:2440–2448. 12027881 10.1046/j.1432-1033.2002.02911.x

[31] Agrawal AA, Petschenka G, Bingham RA, Weber MG, Rasmann S. 2012. Toxic cardenolides: chemical ecology and coevolution of specialized plant-herbivore interactions. New Phytol. 194:28–45. 22292897 10.1111/j.1469-8137.2011.04049.x

[32] Pirahanchi Y, Jessu R, Aeddula NR. 2020. Physiology, sodium potassium pump. Treasure Island (FL): StatPearls Publishing. 30725773

[33] Gonzalez A, Hare JF, Eisner T. 1999. Chemical egg defense in *Photuris* firefly “femmes fatales”. Chemoecology. 9:177–185.

[34] Leimar O, Enquist M, Sillen-Tullberg B. 1986. Evolutionary stability of aposematic coloration and prey unprofitability: a theoretical analysis. Am Nat. 128:469–490.

[35] Yachi S, Higashi M. 1998. The evolution of warning signals. Nature. 394:882–884.

[36] Oba Y, et al 2020. Resurrecting the ancient glow of the fireflies. Sci Adv. 6:eabc5705. 33268373 10.1126/sciadv.abc5705 PMC7710365

[37] Branham MA, Wenzel JW. 2001. The evolution of bioluminescence in cantharoids (Coleoptera: Elateroidea). Fla Entomol. 84:565–586.

[38] Shi G, et al 2012. Age constraint on Burmese amber based on U–Pb dating of zircons. Cretaceous Res. 37:155–163.

[39] Kazantsev S . 2015. *Protoluciola albertalleni* gen. n., sp. n., a new Luciolinae firefly (Insecta: Coleoptera: Lampyridae) from Burmite amber. Russ Entomol J. 24:281–283.

[40] Martin GJ, et al 2019. Higher-level phylogeny and reclassification of Lampyridae (Coleoptera: Elateroidea). Insect Syst Diversity. 3:11.

[41] Zhu C, Xu X, Zhen Y. 2022. Systematic review of the firefly genus *Emeia* Fu, Ballantyne & Lambkin, 2012 (Coleoptera: Lampyridae) from China. Zookeys. 1113:153–166. 36762232 10.3897/zookeys.1113.79721 PMC9848877

[42] Zhu C, Xu X, Zhen Y. 2022. Two new species of *Pyrocoelia* Gorham (Coleoptera: Lampyridae) from Southwest China. Zootaxa. 5162:173–182. 36095513 10.11646/zootaxa.5162.2.6

[43] Berner RA, VandenBrooks JM, Ward PD. 2007. Oxygen and evolution. Science. 316:557–558. 17463279 10.1126/science.1140273

[44] Chen X, et al 2019. Phylogenetic analysis provides insights into the evolution of Asian fireflies and adult bioluminescence. Mol Phylogenet Evol. 140:106600. 31445200 10.1016/j.ympev.2019.106600

[45] Martin GJ . 2020. Advances in the systematics and evolutionary understanding of fireflies (Coleoptera: Lampyridae). Provo (UT): Brigham Young University.

[46] Guo X, et al 2018. Rapid characterization and identification of the chemical constituents and rat metabolites of Deng-Zhan-Xi-Xin injection using ultra high performance liquid chromatography coupled with quadrupole time-of-flight mass spectrometry. J Sep Sci. 41:3569–3582. 30062810 10.1002/jssc.201800470

[47] Zheng W, et al 2017. Rapid characterization of constituents in Tribulus terrestris from different habitats by UHPLC/Q-TOF MS. J Am Soc Mass Spectrom. 28:2302–2318. 28766114 10.1007/s13361-017-1761-5

[48] Rawlinson C, et al 2020. Hierarchical clustering of MS/MS spectra from the firefly metabolome identifies new lucibufagin compounds. Sci Rep. 10:6043. 32269256 10.1038/s41598-020-63036-1 PMC7142086

[49] Zhang S-Q, et al 2018. Evolutionary history of Coleoptera revealed by extensive sampling of genes and species. Nat Commun. 9:205. 29335414 10.1038/s41467-017-02644-4 PMC5768713

[50] Revell LJ . 2012. Phytools: an R package for phylogenetic comparative biology (and other things). Methods Ecol Evol. 2:217–223.

[51] Bollback JP . 2006. SIMMAP: stochastic character mapping of discrete traits on phylogenies. BMC Bioinformatics. 7:88. 16504105 10.1186/1471-2105-7-88 PMC1403802

[52] Li Y, et al 2021. Cretophengodidae, a new cretaceous beetle family, sheds light on the evolution of bioluminescence. Proc R Soc B. 288:20202730. 10.1098/rspb.2020.2730 PMC7893276 33468008

[53] Dolin V . 1980. Click-beetles (Coleoptera, Elateridae) from upper Jurassic of Karatau. Mesozoic fossil insects. Kiev: Naukova Dumka Publishing House; p. 17–81.

[54] Zhen Y, Aardema ML, Medina EM, Schumer M, Andolfatto P. 2012. Parallel molecular evolution in an herbivore community. Science. 337:1634–1637. 23019645 10.1126/science.1226630 PMC3770729

[55] Karageorgi M, et al 2019. Genome editing retraces the evolution of toxin resistance in the monarch butterfly. Nature. 574:409–412. 31578524 10.1038/s41586-019-1610-8 PMC7039281

[56] Wang T, Shi L, Zhen Y. 2022. Gut-specific cardenolide-resistant sodium pump primed an omnivore to feed on toxic oleander. iScience. 25:105616. 36465126 10.1016/j.isci.2022.105616 PMC9713366

[57] Yang L, et al 2023. Predatory fireflies and their toxic firefly prey have evolved distinct toxin resistance strategies. Curr Biol. 33:5160–5168. 37989309 10.1016/j.cub.2023.10.063 PMC10872512

[58] Yang L, et al 2019. Predictability in the evolution of Orthopteran cardenolide insensitivity. Philos Trans R Soc B. 374:20180246. 10.1098/rstb.2018.0246 PMC6560278 31154978

[59] Dubuisson M, Marchand C, Rees JF. 2004. Fireﬂy luciferin as antioxidant and light emitter: the evolution of insect bioluminescence. Luminescence. 19:339–344. 15558801 10.1002/bio.789

[60] Barros MP, Bechara EJ. 1998. Bioluminescence as a possible auxiliary oxygen detoxifying mechanism in elaterid larvae. Free Radical Biol Med. 24:767–777. 9586807 10.1016/s0891-5849(97)00335-3

[61] Berner RA . 2006. GEOCARBSULF: a combined model for Phanerozoic atmospheric O_2_ and CO_2_. Geochim Cosmochim Acta. 70:5653–5664.

[62] Slater SM, Twitchett RJ, Danise S, Vajda V. 2019. Substantial vegetation response to early Jurassic global warming with impacts on oceanic anoxia. Nat Geosci. 12:462–467.

[63] Freeman MR, Doherty J. 2006. Glial cell biology in *Drosophila* and vertebrates. Trends Neurosci. 29:82–90. 16377000 10.1016/j.tins.2005.12.002

[64] Petschenka G, Pick C, Wagschal V, Dobler S. 2013. Functional evidence for physiological mechanisms to circumvent neurotoxicity of cardenolides in an adapted and a non-adapted hawk-moth species. Proc R Soc B. 280:20123089. 10.1098/rspb.2012.3089 PMC3619502 23516239

[65] Torrie LS, et al 2004. Resolution of the insect ouabain paradox. Proc Natl Acad Sci U S A. 101:13689–13693. 15347816 10.1073/pnas.0403087101 PMC518814

[66] Groen SC, et al 2017. Multidrug transporters and organic anion transporting polypeptides protect insects against the toxic effects of cardenolides. Insect Biochem Mol Biol. 81:51–61. 28011348 10.1016/j.ibmb.2016.12.008 PMC5428987

[67] Day JC . 2011. Parasites, predators and defence of fireflies and glow-worms. Lampyrid. 1:70–102.

[68] Okada YK . 1928. Two Japanese aquatic glowworms. Trans R Entomol Soc Lond. 76:101–108.

[69] Fu X, et al 2007. Structure and function of the eversible glands of the aquatic firefly *Luciola leii* (Coleoptera: Lampyridae). Chemoecology. 17:117–124.

[70] Al-Wathiqui N, Fallon TR, South A, Weng J-K, Lewis SM. 2016. Molecular characterization of firefly nuptial gifts: a multi-omics approach sheds light on postcopulatory sexual selection. Sci Rep. 6:38556. 28004739 10.1038/srep38556 PMC5177949

[71] Jeng ML, Yang PS, Engel MS. 2007. The firefly genus *Vesta* in Taiwan (Coleoptera: Lmpyridae). J Kans Entomol Soc. 80:265–280.

[72] Fu XH, Ballantyne L, Lambkin C. 2012. *Emeia* gen. nov., a new genus of Luciolinae fireflies from China (Coleoptera: Lampyridae) with an unusual trilobite-like larva, and a redescription of the genus Curtos Motschulsky. Zootaxa. 3403:1–53.

[73] Laslett D, Canbäck B. 2008. ARWEN: a program to detect tRNA genes in metazoan mitochondrial nucleotide sequences. Bioinformatics. 24:172–175. 18033792 10.1093/bioinformatics/btm573

[74] Nurk S, Meleshko D, Korobeynikov A, Pevzner PA. 2017. metaSPAdes: a new versatile metagenomic assembler. Genome Res. 27:824–834. 28298430 10.1101/gr.213959.116 PMC5411777

[75] Allio R, et al 2020. MitoFinder: efficient automated large-scale extraction of mitogenomic data in target enrichment phylogenomics. Mol Ecol Resour. 20:892–905. 32243090 10.1111/1755-0998.13160 PMC7497042

[76] Andrews S . 2010. FastQC: a quality control tool for high throughput sequence data. http://www.bioinformatics.babraham.ac.uk/projects/fastqc/.

[77] Grabherr MG, et al 2011. Full-length transcriptome assembly from RNA-Seq data without a reference genome. Nat Biotechnol. 29:644–652. 21572440 10.1038/nbt.1883 PMC3571712

[78] Bolger AM, Lohse M, Usadel B. 2014. Trimmomatic: a flexible trimmer for illumina sequence data. Bioinformatics. 30:2114–2120. 24695404 10.1093/bioinformatics/btu170 PMC4103590

[79] Rosnack KJ, Reid MJ, Ladak A, Cleland G. 2016. Screening solution using the software platform UNIFI: an integrated workflow by waters. Washington (DC): ACS Publications. p. 155–172.

[80] Li W, Godzik A. 2006. Cd-hit: a fast program for clustering and comparing large sets of protein or nucleotide sequences. Bioinformatics. 22:1658–1659. 16731699 10.1093/bioinformatics/btl158

[81] Fu L, Niu B, Zhu Z, Wu S, Li W. 2012. CD-HIT: accelerated for clustering the next-generation sequencing data. Bioinformatics. 28:3150–3152. 23060610 10.1093/bioinformatics/bts565 PMC3516142

[82] Emms DM, Kelly S. 2015. OrthoFinder: solving fundamental biases in whole genome comparisons dramatically improves orthogroup inference accuracy. Genome Biol Evol. 16:1–14. 10.1186/s13059-015-0721-2 PMC4531804 26243257

[83] Katoh K, Standley DM. 2013. MAFFT multiple sequence alignment software version 7: improvements in performance and usability. Mol Biol Evol. 30:772–780. 23329690 10.1093/molbev/mst010 PMC3603318

[84] Abascal F, Zardoya R, Telford MJ. 2010. Translatorx: multiple alignment of nucleotide sequences guided by amino acid translations. Nucleic Acids Res. 38:W7–W13. 20435676 10.1093/nar/gkq291 PMC2896173

[85] Castresana J . 2000. Selection of conserved blocks from multiple alignments for their use in phylogenetic analysis. Mol Biol Evol. 17:540–552. 10742046 10.1093/oxfordjournals.molbev.a026334

[86] Talavera G, Castresana J. 2007. Improvement of phylogenies after removing divergent and ambiguously aligned blocks from protein sequence alignments. Syst Biol. 56:564–577. 17654362 10.1080/10635150701472164

[87] Lanfear R, Calcott B, Ho SY, Guindon S. 2012. PartitionFinder: combined selection of partitioning schemes and substitution models for phylogenetic analyses. Mol Biol Evol. 29:1695–1701. 22319168 10.1093/molbev/mss020

[88] Lanfear R, Calcott B, Kainer D, Mayer C, Stamatakis A. 2014. Selecting optimal partitioning schemes for phylogenomic datasets. BMC Evol Biol. 14:82. 24742000 10.1186/1471-2148-14-82 PMC4012149

[89] Stamatakis A . 2014. RAxML version 8: a tool for phylogenetic analysis and post-analysis of large phylogenies. Bioinformatics. 30:1312–1313. 24451623 10.1093/bioinformatics/btu033 PMC3998144

[90] Kundrata R, Packova G, Prosvirov AS, Hoffmannova J. 2021. The fossil record of Elateridae (Coleoptera: Elateroidea): described species, current problems and future prospects. Insects. 12:286. 33805978 10.3390/insects12040286 PMC8064311

[91] Reis M, Yang Z. 2011. Approximate likelihood calculation on a phylogeny for Bayesian estimation of divergence times. Mol Biol Evol. 28:2161–2172. 21310946 10.1093/molbev/msr045

[92] Altschul SF, Gish W, Miller W, Myers EW, Lipman DJ. 1990. Basic local alignment search tool. J Mol Biol. 215:403–410. 2231712 10.1016/S0022-2836(05)80360-2

[93] Kearse M, et al 2012. Geneious basic: an integrated and extendable desktop software platform for the organization and analysis of sequence data. Bioinformatics. 28:1647–1649. 22543367 10.1093/bioinformatics/bts199 PMC3371832

[94] Edgar RC . 2004. MUSCLE: multiple sequence alignment with high accuracy and high throughput. Nucleic Acids Res. 32:1792–1797. 15034147 10.1093/nar/gkh340 PMC390337

[95] Yang Z . 2007. PAML 4: phylogenetic analysis by maximum likelihood. Mol Biol Evol. 24:1586–1591. 17483113 10.1093/molbev/msm088

[96] Laursen M, Gregersen JL, Yatime L, Nissen P, Fedosova NU. 2015. Structures and characterization of digoxin-and bufalin-bound Na+, K+-ATPase compared with the ouabain-bound complex. Proc Natl Acad Sci U S A. 112:1755–1760. 25624492 10.1073/pnas.1422997112 PMC4330780

[97] Vilar S, Cozza G, Moro S. 2008. Medicinal chemistry and the molecular operating environment (MOE): application of QSAR and molecular docking to drug discovery. Curr Top Med Chem. 8:1555–1572. 19075767 10.2174/156802608786786624

[98] Eswar N, et al 2007. Comparative protein structure modeling using MODELLER. Curr Protoc Protein Sci. 50:5–6. 10.1002/0471140864.ps0209s50 18429317

[99] Jumper J, et al 2021. Highly accurate protein structure prediction with AlphaFold. Nature. 596:583–589. 34265844 10.1038/s41586-021-03819-2 PMC8371605

[100] DeLano WL. 2002. PyMOL: an open-source molecular graphics tool. *CCP4 Newsl. Protein Crystallogr.* 40:82–92.

[101] Petschenka G, et al 2013. Stepwise evolution of resistance to toxic cardenolides via genetic substitutions in the Na+/K+-ATPase of milkweed butterflies (Lepidoptera: Danaini). Evolution. 67:2753–2761. 24033181 10.1111/evo.12152

