# Supplementary material for: Firefly toxin lucibufagins evolved after the origin of bioluminescence
Source: PNAS Nexus. 2024 Jun 25;3(6):pgae215. doi: 10.1093/pnasnexus/pgae215 (PMC11197309; doi:10.1093/pnasnexus/pgae215)
Supplement: pgae215_Supplementary_Data [file pgae215_supplementary_data.pdf]

Supplementary Information for

Firefly toxin lucibufagins evolved after the origin of bioluminescence

Authors

Chengqi Zhu[1,2,3], Xiaoli Lu[1,2,3], Tianlong Cai[1], Kangli Zhu[1], Lina Shi[1,2,3], Yinjuan Chen[4], Tianyu Wang[1,2,3], Yaoming Yang[1,2,3], Dandan Tu[1,2,3], Qi Fu[1,2,3], Jing Huang[1,2,3], Ying Zhen[1,2,3]*

Affiliations

[1] Westlake Laboratory of Life Sciences and Biomedicine, Hangzhou, Zhejiang, China.

[2] Key Laboratory of Structural Biology of Zhejiang Province, School of Life Sciences, Westlake University, Hangzhou, Zhejiang, China.

[3] Institute of Biology, Westlake Institute for Advanced Study, Hangzhou, Zhejiang, China.

[4] Instrumentation and Service Center for Molecular Sciences, Westlake University, Hangzhou, Zhejiang, China.

* zhenying@westlake.edu.cn

This file includes:

Figures S1 to S10
Tables S1 to S11
SI References

Supplemental Figures

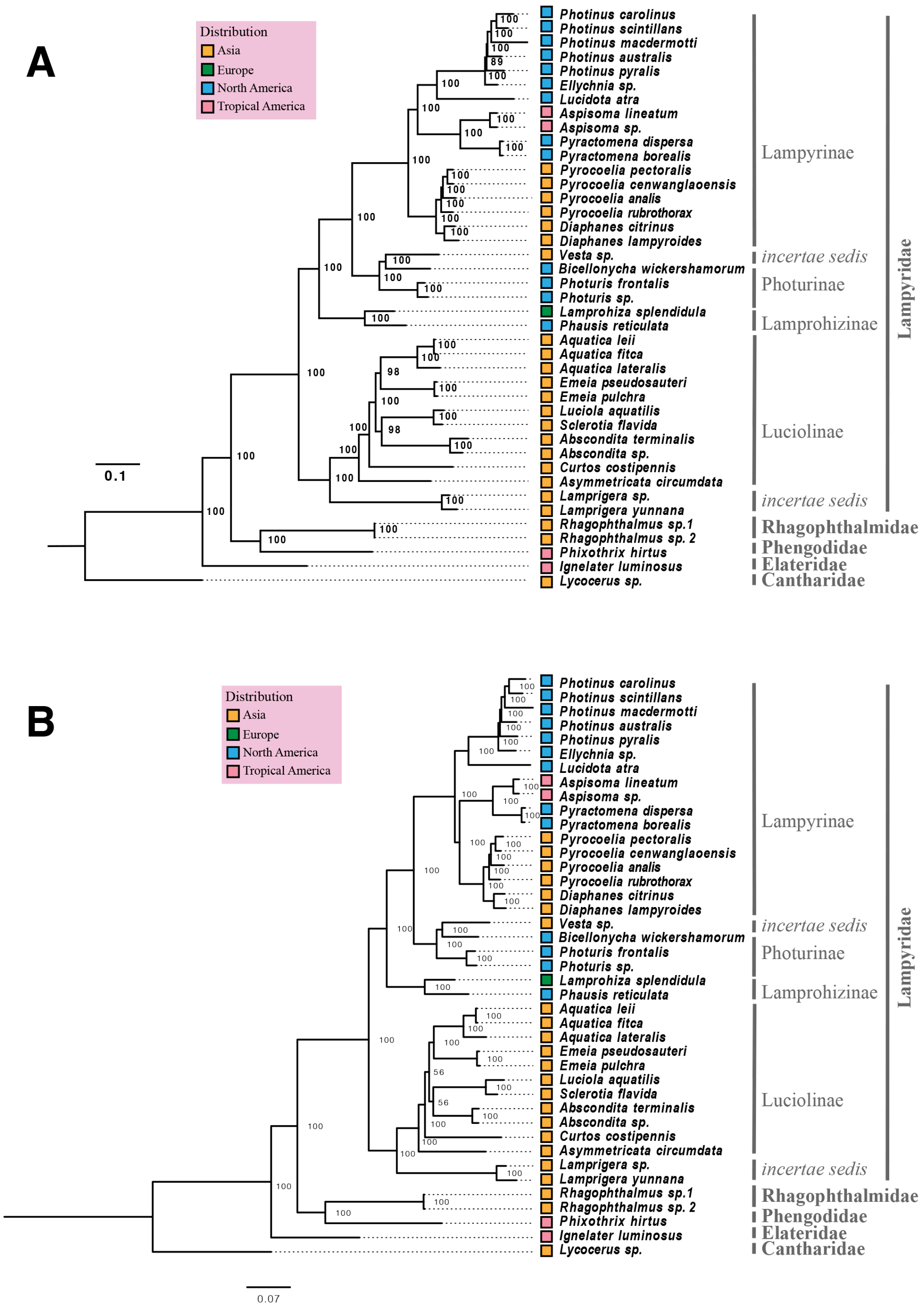

Fig. S1. Phylogenetic trees of bioluminescent beetles. Maximum likelihood trees of the 41 species inferred from concatenated nucleotide sequences (A) or concatenated protein sequences (B) of 1,353 single copy orthologs. The nucleotide tree used GTRGAMMAI model and 1,000 bootstrap replications. The protein tree used PROTGAMMALGX model and 100 bootstrap replications. The trees were rooted with the non-bioluminescent outgroup *Lycocerus* sp. in the Cantharidae family. Colored squares denote the geographic distribution of each species.

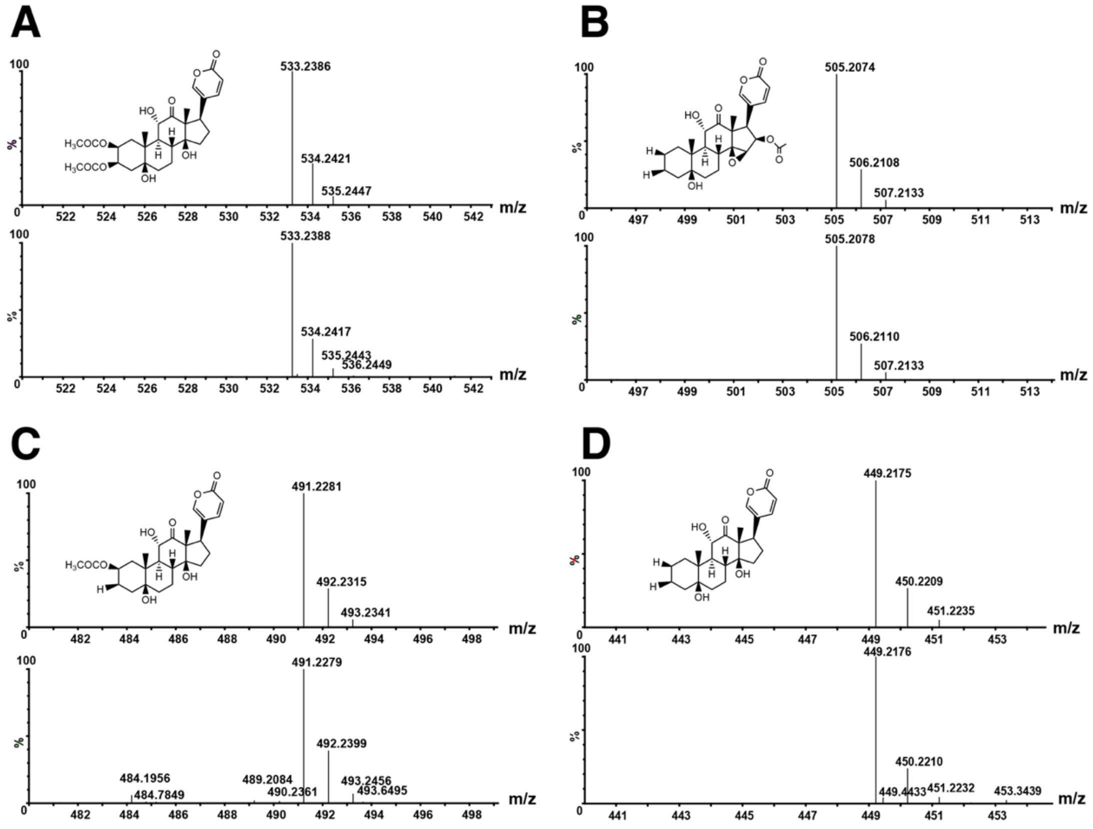

Fig. S2. Theoretical (upper panel) and experimental (lower panel) mass spectra of lucibufagin C $[M+H]^+$ (A), lucibufagin J $[M+H]^+$ (B), lucibufagin D/E $[M+H]^+$ (C) and core lucibufagin $[M+H]^+$ (D). Theoretical and experimental spectra match well with precise m/z value and isotope peak distributions.

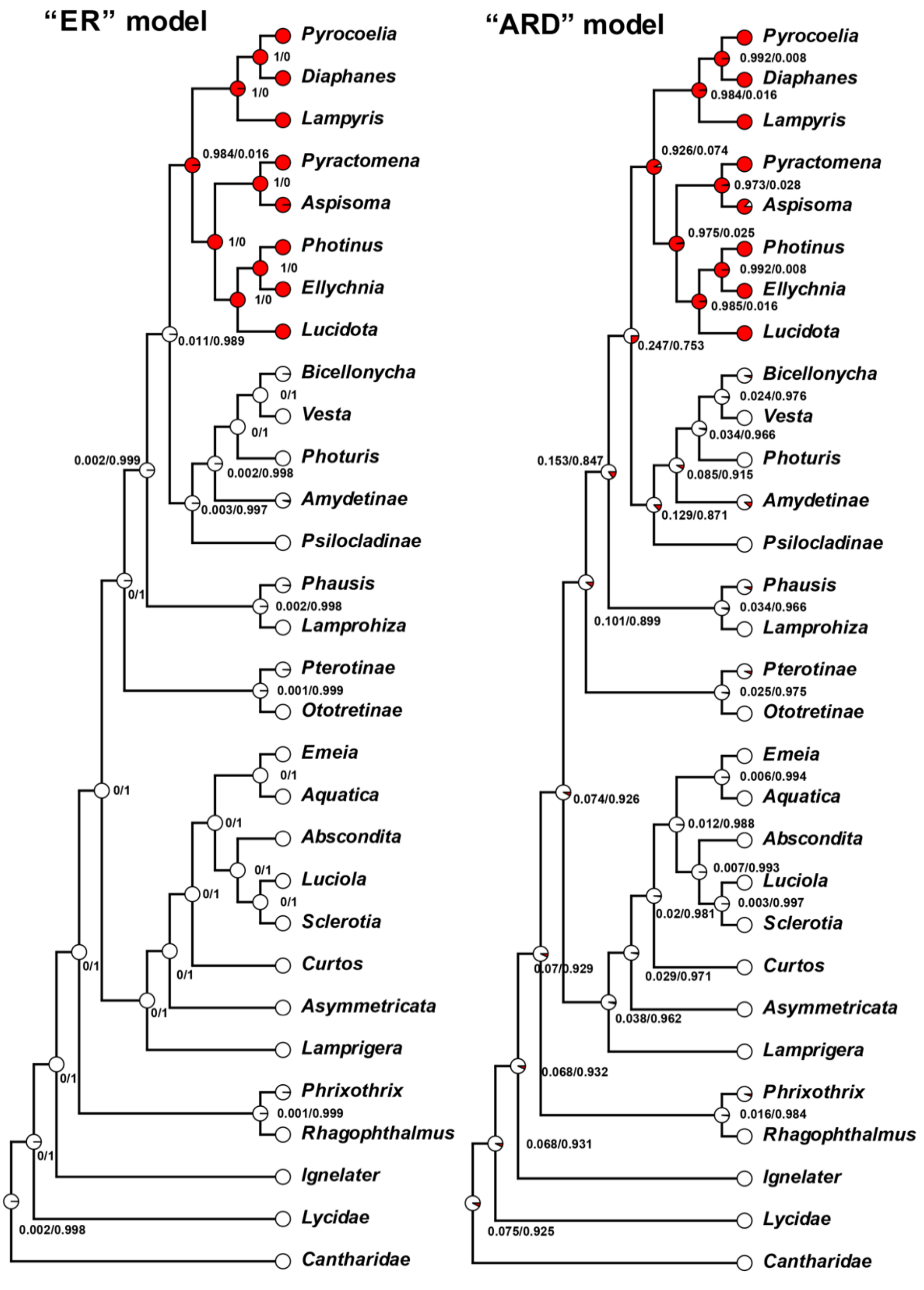

Fig. S3. Ancestral state reconstructions of the LBGs across the bioluminescent beetles under "ER" model (left) and "ARD" model (right) using phytools. Inferred ancestral states are shown by pie charts, and the red and white portions denote the posterior probabilities of LBGs presence and absence, respectively.

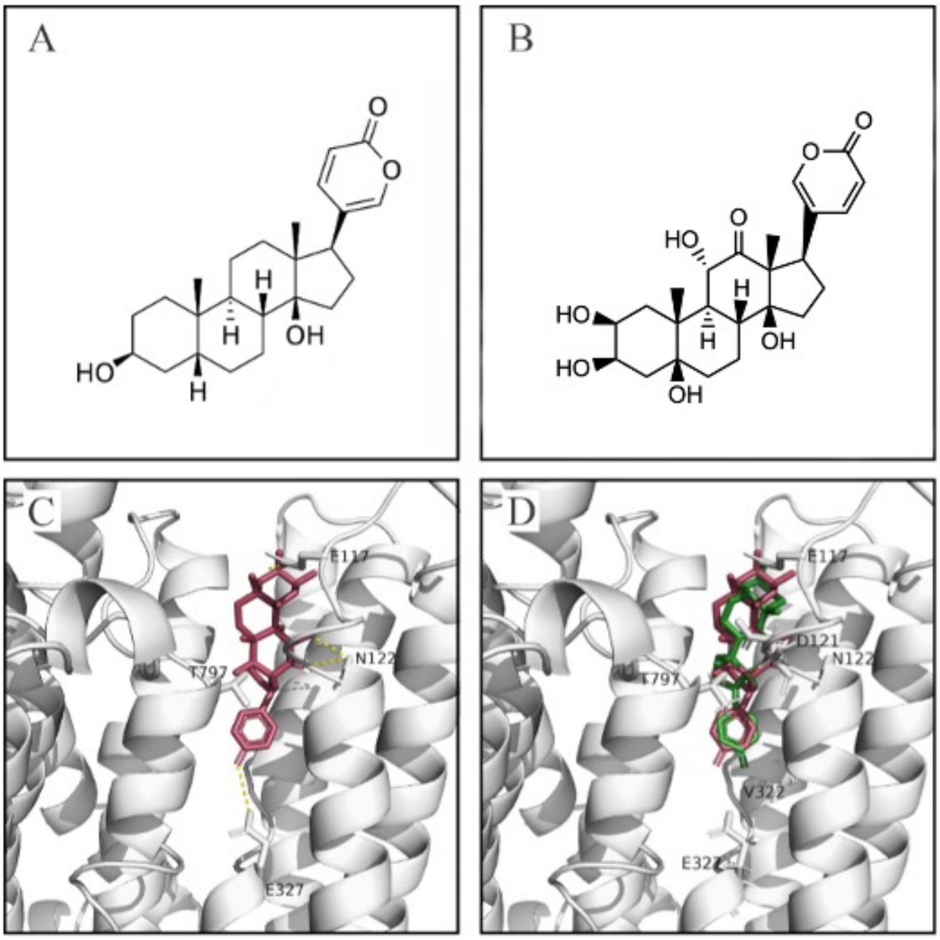

Fig. S4. Docking structure of core lucibufagin and bufalin on pig ATP1A1. Chemical structures of (A) bufalin and (B) core lucibufagin. The best docking structure of core lucibufagin-ATP1A1 complex, defined as the structure from the top 10 highest affinity dockings that was closest to the co-crystal coordinates of bufalin, shown only with core lucibufagin (C), or with both core lucibufagin and bufalin (D). The β-surface of core lucibufagin interacts with residues E117, E327, N122 and T797. Bufalin interacts with residues D121, E327, V322 and T797. Red: core lucibufagin; green: bufalin; yellow dotted lines: hydrogen bounds.

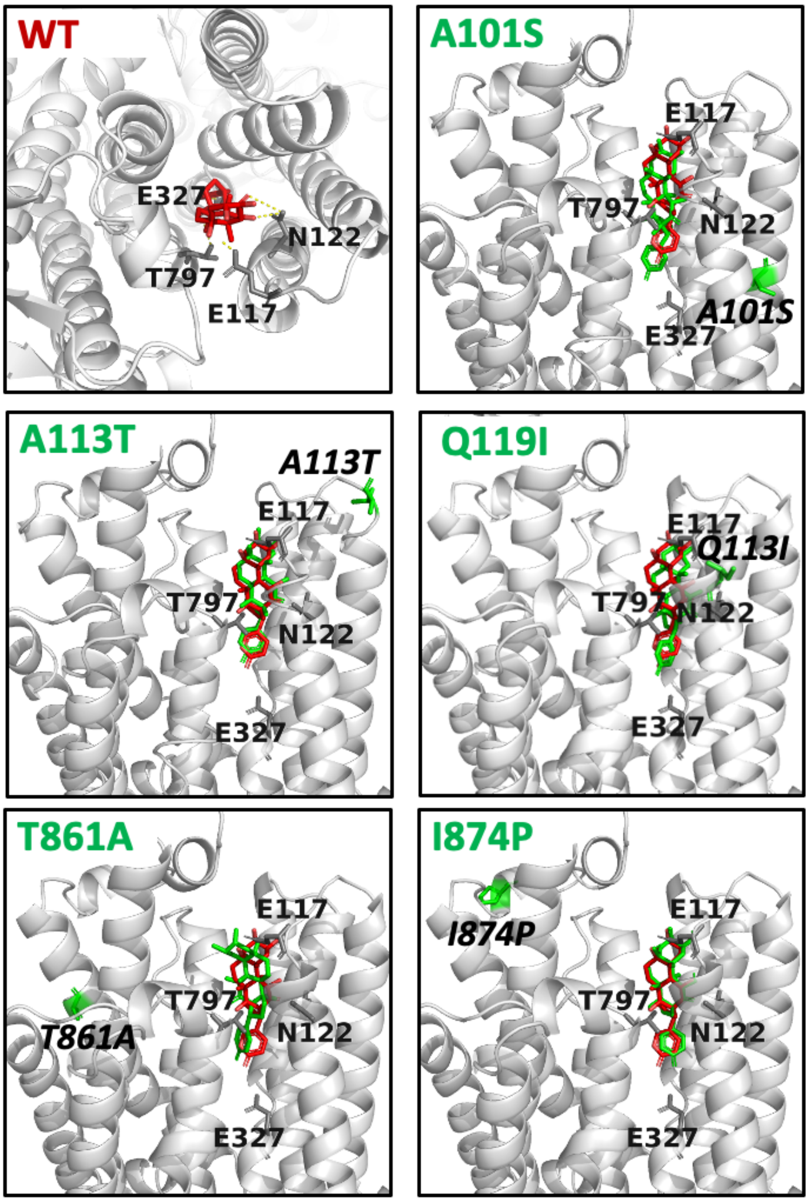

Fig. S5. Structures of core lucibufagin binding pocket and molecular docking simulations of pig ATP1A1 carrying single amino acid substitution. (WT) pig ATP1A1 bound to core lucibufagin (in red). The docking position was inferred by the bufalin and pig ATP1A1 co-crystal structure (PDB: 4RES). Yellow dotted lines show possible hydrogen bonds between ATP1A1 and core lucibufagin. All ligand-protein interactions formed on the β-surface of core lucibufagin and bufalin are listed in Table S9.

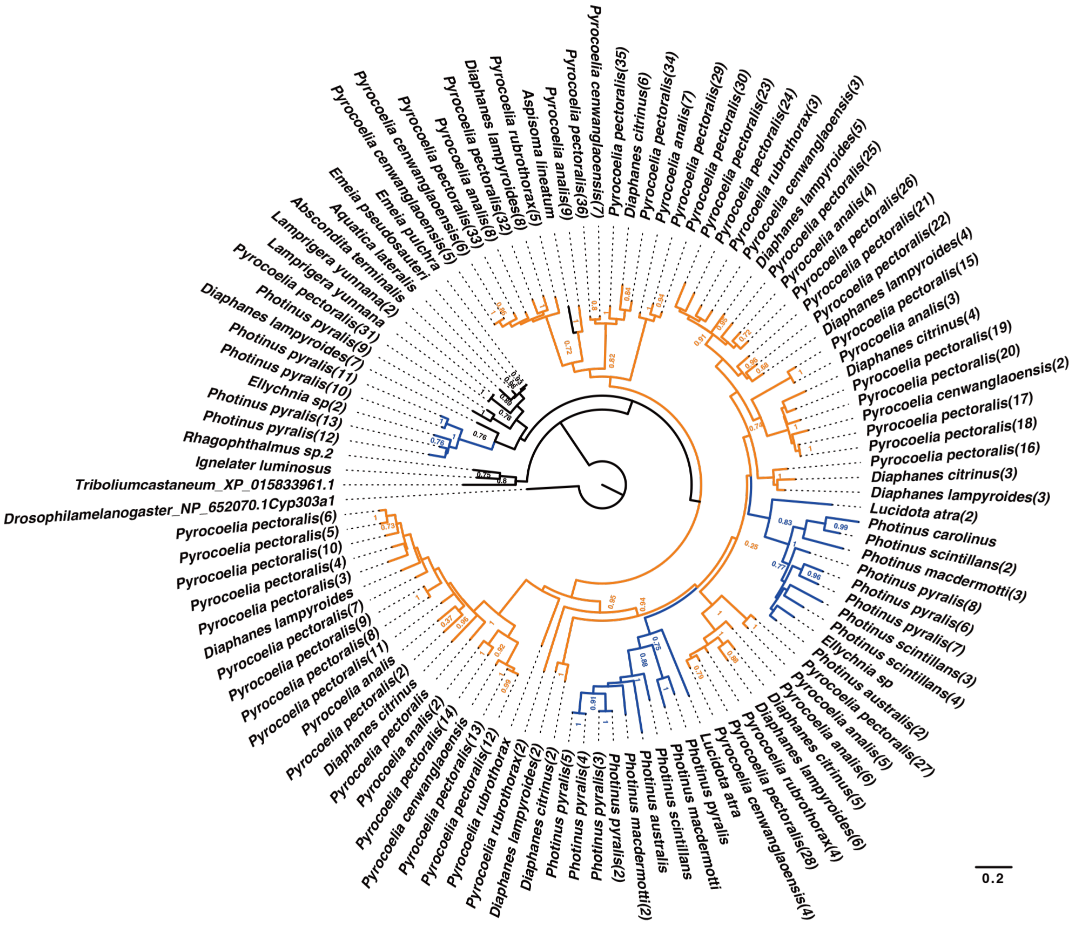

Fig. S6. Maximum likelihood tree of CYP303 from 41 beetles suggests gene family expansion in LBGs-containing species (highlighted in colors). The fruit fly *D. melanogaster* CYP303 was used as an outgroup. ML Bootstrap values greater than 0.7 from 1,000 replicates are shown.

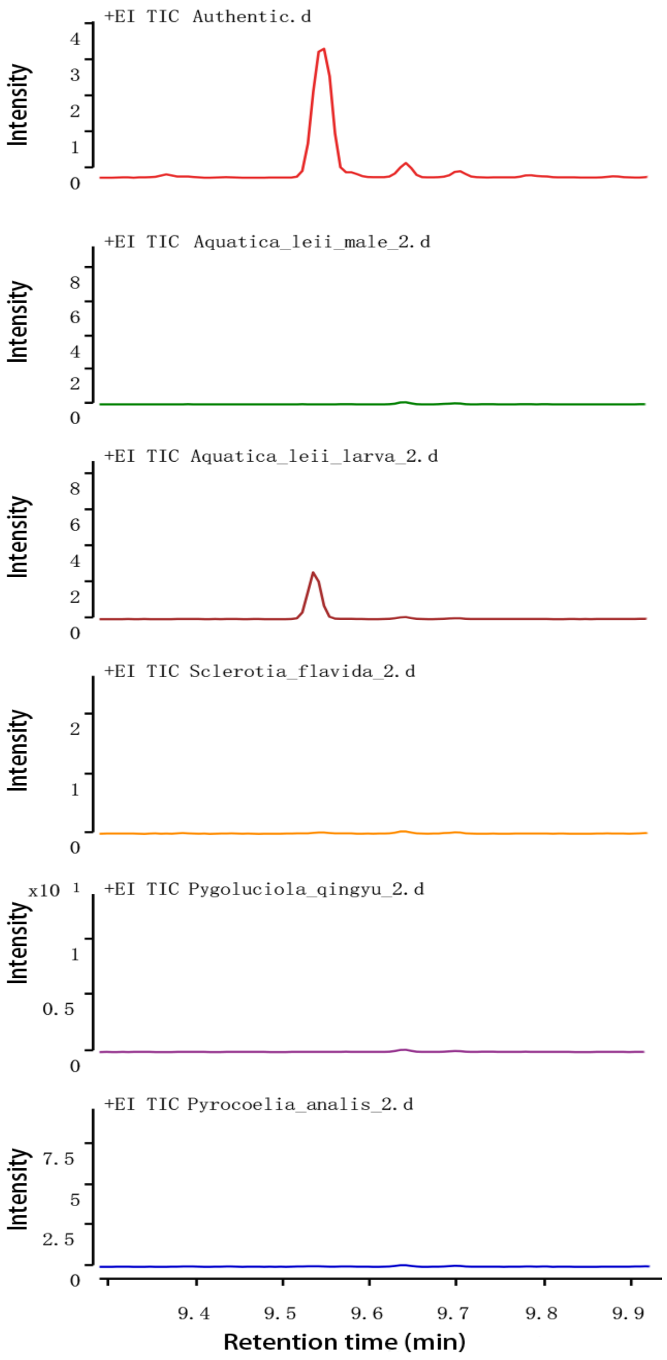

Fig. S7. GC-MS analysis of terpinolene in firefly species. The chromatograms from top to bottom represent authentic terpinolene standard, extracts from adult male of *Aquatica leii*, larva of *A. leii*, *Sclerotia flavida*, *Pygoluciola qingyu* and *Pyrocoelia analis*, respectively. Terpinolene were only detected in larva of *A. leii*.

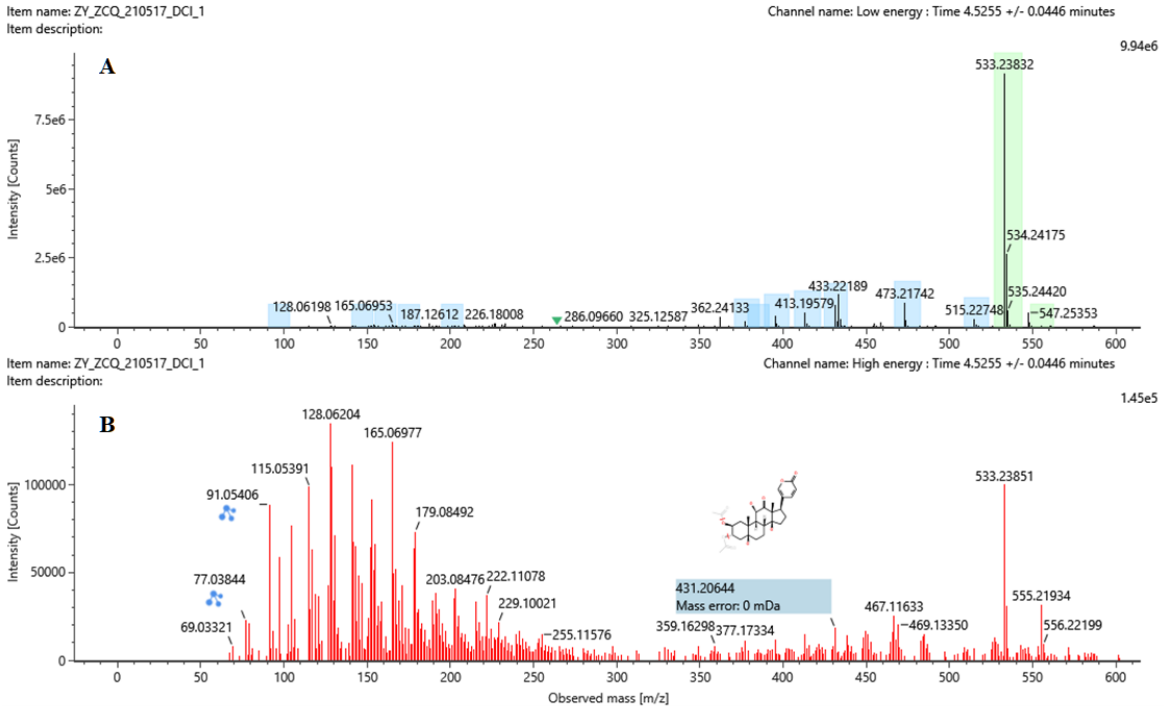

Fig. S8. Parent ion of lucibufagin C and its fragments in mass spectra from *Diaphanes citrinus* sample. A. low energy channel; B. high energy channel. MS peak at m/z=533.2385 Da is attributed to protonated lucibufagin C ($[C_{28}H_{37}O_{10}]^{+}$). Other marked MS peaks are fragments of $[C_{28}H_{37}O_{10}]^{+}$.

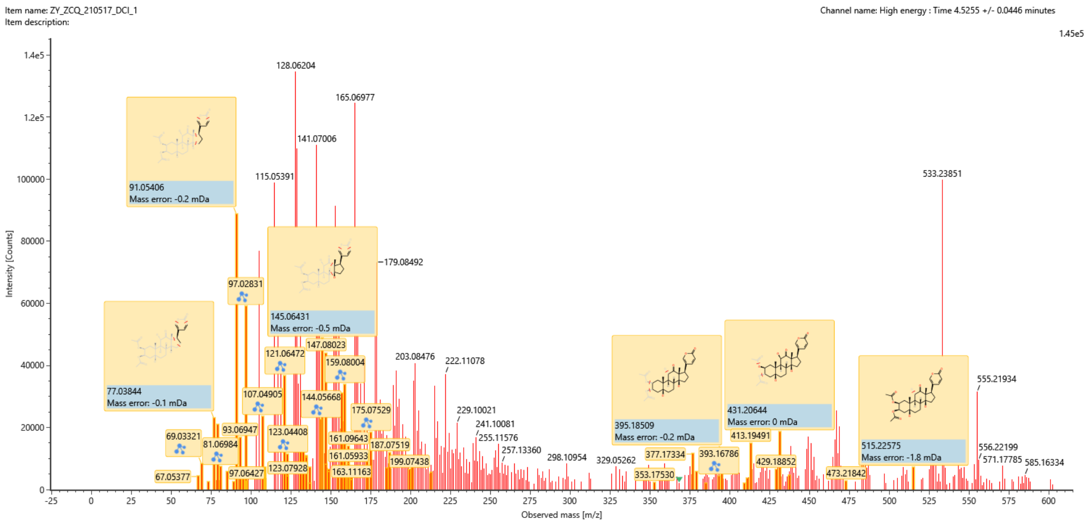

Fig. S9. Fragment assignment of protonated lucibufagin C from *Diaphanes citrinus* sample in high energy mass spectrum using UNIFI.

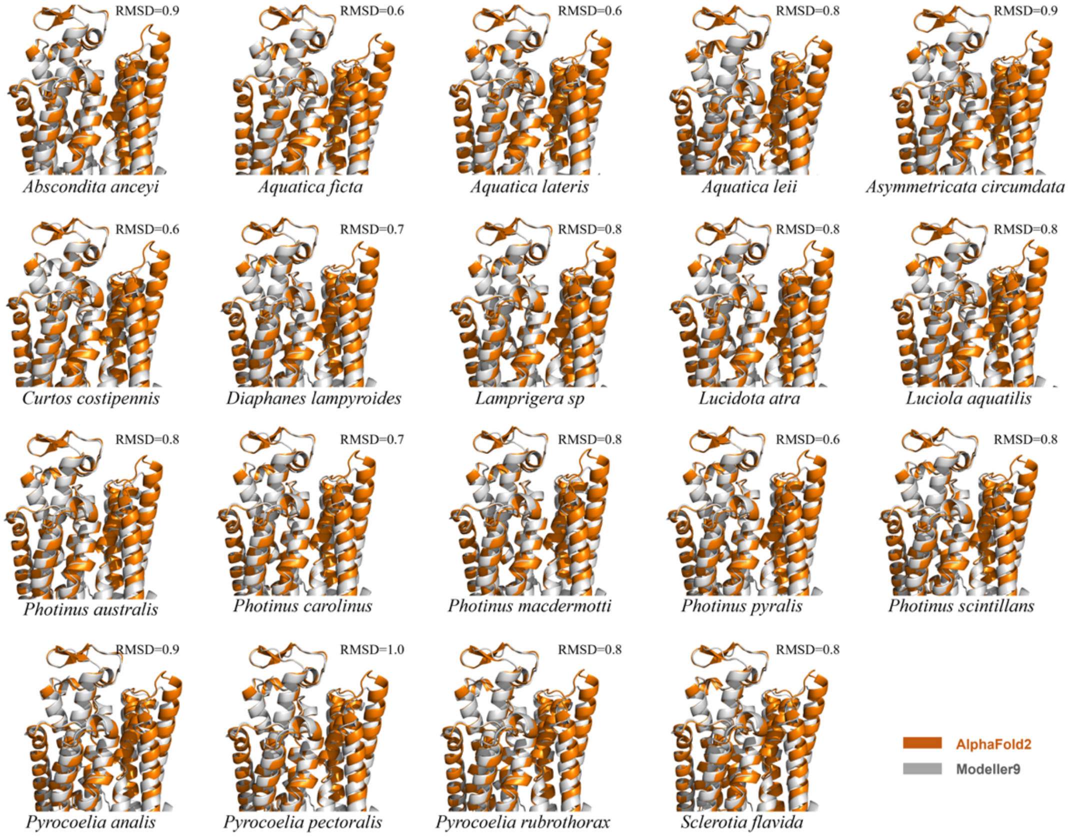

Fig. S10. Structural alignment of predicted structures of native ATPα proteins from firefly species using AlphaFold2 and Modeller9, measured as heavy-atom root mean square deviation (RMSD) in Ångstrom.

Supplemental Tables

Table S1. Sampling information of the 21 species collected in our study.

Family/subfamily	Species	Sample info	Location
Lampyridae/Lampyrinae	*Pyrocoelia rubrothorax*[1]	Adult male	Baise, Guangxi, China
	Pyrocoelia analis	Adult male	Tunchang, Hainan, China
	Pyrocoelia pectoralis[3]	Adult male	Xinyang, Henan, China
	Pyrocoelia cenwanglaoensis[1]	Adult male	Nanning, Guangxi, China
	Diaphanes lampyroides	Adult male	Nanning, Guangxi, China
	Diaphanes citrinus	Adult male	Nanning, Guangxi, China
Lampyridae/Luciolinae	*Aquatica leii*	Adult male	Ningdu, Jiangxi, China
	Aquatica ficta	Adult male	Ningdu, Jiangxi, China
	Abscondita sp.	Adult male	Hangzhou, Zhejiang, China
	Curtos costipennis	Adult male	Fengxian, Shanghai, China
	Sclerotia flavida	Adult male	Tunchang, Hainan, China
	Asymmetricata circumdata[3]	Adult male	Tunchang, Hainan, China
	Emeia pseudosauteri	Adult male	Leshan, Sichuan, China
	Emeia pulchra[1]	Adult male	Lishui, Zhejiang, China
Lampyridae/*incertae sedis*	*Lamprigera* sp.	Larva	Binhai, Tianjing, China
	Vesta sp.	Adult male	Shaoguan, Guangdong, China
	Vesta sp.2[2]	Adult male	Rong'an, Guangxi, China
Lampyridae/Ototretinae	*Stenocladius* sp.[2]	Adult male	Jianfengling, Hainan, China
Lampyrinae/Psilocladiane	*Cyphonocerus* sp.[2]	Adult male	Jianfengling, Hainan, China
Rhagophthamidae	*Rhagophthalmus* sp. 2	Adult female	Tunchang, Hainan, China
Cantharidae	*Lycocerus* sp.	Adult male	Shaoguan, Guangdong, China

[1]three newly identified species

[2]preserved museum specimens obtained from the museum of biology, Sun Yat-sen University

[3]two species with publicly available genomic data.

Table S2. Summary of data from 41 taxa used in our study.

Family/ subfamily	Species	Data type	Tissue	Data source	Reference
Lampyridae/ Lampyrinae	*Pyrocoelia rubrothorax*	T	Whole body	SRR22498553	this study
	Pyrocoelia analis	T	Whole body	SRR22498552	
	Pyrocoelia pectoralis	G	Whole body	GigaDB	(1)
	Pyrocoelia cenwanglaoensis	T	Whole body	SRR22498543	
	Diaphanes lampyroides	T	Whole body	SRR22498542	
	Diaphanes citrinus	T	Whole body	SRR22498541	
	Photinus australis	T	Head	SRR2103451	(2)
	Photinus pyralis	G	Whole body	Fireflybase	(3)
	Photinus carolinus	T	Head	SRR2103825	(2)
	Photinus scintillans	T	Head	SRR2104146	(2)
	Photinus macdermotti	T	Head	SRR2103834	(2)
	Ellychnia sp.	T	Whole body	SRR14833222	(4)
	Aspisoma lineatum	T	Lantern	SRR4407797	(5)
	Aspisoma sp.	T	Abdomen	SRR14833225	(4)
	Pyractomena borealis	T	Head	SRR2104393	(2)
	Pyractomena dispersa	T	Abdomen	SRR14833228	(4)
	Lucidota atra	T	Head	SRR2103572	(2)
	Lamprohiza splendidula	T	Whole body	SRR2083681	(6)
	Phausis reticulata	T	Head	SRR2103663	(2)
Lampyridae/ Photurinae	*Photuris frontalis*	T	Head	SRR2104391	(2)
	Photuris sp.	T	Head	SRR2104392	(2)
	Bicellonycha wickershamorum	T	Whole body	SRR14833220	(4)
Lampyridae/ *incertae sedis*	*Vesta sp.*	T	Thorax	SRR22498540	
	Lamprigera sp.	T	Head	SRR22498537	
		T	Thorax	SRR22498538	
		T	Abdomen	SRR22498539	
	Lamprigera yunnana	G	Whole body	GCA_013368075.1	(7)
Lampyridae/ Luciolinae	*Asymmetricata circumdata*	T	Whole body	SRR4045938	
	Curtos costipennis	T	Whole body	SRR22498536	
	Sclerotia flavida	T	Whole body	SRR22498551	
	Aquatica lateralis	G	Whole body	Fireflybase	(3)
	Aquatica leii	T	Whole body	SRR22498550	
	Aquatica ficta	T	Whole body	SRR22498549	
	Luciola aquatilis	T	Whole body	SRR3195455	(8)
	Emeia pseudosauteri	T	Whole body	SRR22498548	
	Emeia pulchra	T	Whole body	SRR22498547	
	Abscondita sp.	T	Whole body	SRR22498546	
	Abscondita terminalis	G	Whole body	GCA_013368085.1	(7)
Phengodidae	*Phrixothrix hirtus*	T	Lantern	SRR4413772	(9)
Rhagophthamidae	*Rhagophthalmus sp.1*	T	Whole body	SRR4045941	(10)
	Rhagophthalmus sp.2	T	Whole body	SRR22498545	
Elateridae	*Ignelater luminosus*	G	Whole body	Fireflybase	(3)
Cantharidae	*Lycocerus sp.*	T	Abdomen	SRR22498544	

T: Transcriptome, G: Genome

Fireflybase: http://www.fireflybase.org/firefly_data.html

GigaDB: http://gigadb.org/dataset/view/id/100376/File_page/

Table S3. Summary statistics of *de novo* assembled transcriptomes. A. Samples sequenced in this study. B. Samples with publicly available RNAseq data.

A.

Family/ Subfamily	Species	Number of raw reads (pairs)	Trinity assemblies					
			%GC	Total of Trinity transcripts	Total of longest isoforms	Contig N50	Median contig length (bp)	Mean contig length (bp)
Lampyridae/ Lampyrinae	*Diaphanes lampyroides*	21,705,939	37.29	87,352	15,908	2,554	590	1,279.68
	Pyrocoelia cenwanglaoensis	19,869,347	36.16	107,069	15,841	2,864	593	1,374.07
	Pyrocoelia analis	19,516,221	37.28	123,865	25,919	2,657	528	1,249.36
	Pyrocoelia rubrothorax	22,166,767	35.93	117.305	16,974	2,887	523	1,310.12
	Diaphane citrinus	18,520,012	36.58	126,183	17,599	2,839	542	1,309.45
Lampyridae/ Luciolinae	*Aquatica leii*	11,675,393	35.49	74,918	16,553	2,565	524	1,216.52
	Aquatica fitca	11,564,829	35.63	74,053	15,608	2,686	512	1,240.58
	Abscondita sp.	10,820,638	35.76	50,592	13,855	3,176	660	1,514.17
	Curtos costipennis	10,147,858	35.56	67,811	15,049	2,520	673	1,332.10
	Sclerotia flavida	9,640,088	36.39	51,976	14,297	2,287	514	1,141.94
	Emeia pseudosauteri	19,968,276	36.68	107,462	16,321	2,832	519	1,286.24
	Emeia pulchra	23,366,902	36.70	96,278	15,947	2,912	522	1,323.10
Lampyridae/ *incertae sedis*	*Lamprigera sp.*	19,973,725	34.80	178,927	22,161	2,755	452	1,156.68
	Vesta sp.	15,346,025	35.13	63,568	18,010	1,570	413	837.41
Rhagophthamidae	*Rhagophthalmus sp. 2*	22,192,747	34.91	86,901	14,249	3,346	605	1,518.73
Cantharidae	*Lycocerus sp.*	15,346,025	35.14	66,185	14,327	2,668	486	1,206.60

B.

Family/ Subfamily	Species	Number of raw reads (pairs)	Trinity assemblies					
			%GC	Total of Trinity transcripts	Total of longest isoforms	Contig N50	Median contig length (bp)	Mean contig length (bp)
Lampyridae/ Lampyrinae	*Ellychnia sp.*	18,693,494	40.26	52,388	16,464	2,376	647	1,278.59
	Photinus australis	13,145,094	39.61	49,343	15,536	2,108	602	1,149.93
	Photinus carolinus	7,472,450	39.31	43,478	14,131	2,394	613	1,256.04
	Photinus scintillans	15,456,064	39.68	68,737	16,019	2,589	557	1,257.63
	Photinus macdermotti	15,851,638	40.91	46,283	13,864	2,686	774	1,439.34
	Aspisoma lineatum	27,191,053	38.03	74,375	16,454	2,775	476	1,239.90
	Aspisoma sp.	8,870,660	40.26	30.293	13,336	1,650	555	979.74
	Pyractomena borealis	10,575,315	39.71	47,815	13,401	2,522	591	1,265.93
	Pyrocoelia dispersa	6,415,590	39.50	31.124	12,954	1,559	497	907.50
	Lucidota atra	11,194,970	38.95	59,702	13,810	2,533	623	1,301.07
	Lamprohiza splendidula	15,226,754	36.74	59,658	18,607	1,547	474	893.69
	Phausis reticulata	9,245,174	36.60	46,272	15,329	2,450	523	1,190.62
Lampyridae/ Photurinae	*Photuris frontalis*	11,373,006	34.63	57,948	15,123	2,356	556	1,200.93
	Photuris sp.	12,359,044	34.43	56,586	13,945	2,605	590	1,298.89
	Bicellonycha wickershamorum	13,623,424	34.14	49.481	15,285	2.046	558	1,107.54
Lampyridae/ Luciolinae	*Asymmetricata circumdata*	35,979,167	35.69	37,718	16,215	1,794	508	1,000.39
	Luciola aquatilis	31,766,634	35.49	30,343	12,876	2,292	587	1,216.75
Phengodidae	*Phrixothrix hirtus*	10,624,452	37.02	21,349	9,143	877	369	616.53
Rhagophthamidae	*Rhagophthalmus sp. 1*	29,909,815	37.02	28,984	12,939	2,596	642	1,339.88

Table S4. Precursor ion and common fragment ions analysis based on eight known LBG skeleton structures.

Structure	Component	Group		Precursor ion		Characteristic fragment ion		
		R1	R2	$[M+H]^+$	m/z	Fragment loss	$[M+H]^+$	m/z
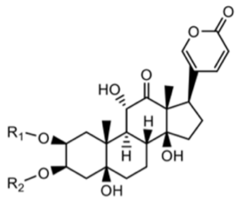	lucibufagin C	$-COCH_3$	$-COCH_3$	$C_{28}H_{37}O_{10}$	533.2386	$-C_4H_6O_3$<br>$-C_4H_6O_3-H_2O$<br>$-C_4H_6O_3-2H_2O$<br>$-C_4H_6O_3-3H_2O$	$C_{24}H_{31}O_7$<br>$C_{24}H_{29}O_6$<br>$C_{24}H_{27}O_5$<br>$C_{24}H_{25}O_4$	431.2070<br>413.1964<br>395.1858<br>377.1753
	lucibufagin D	$-COCH_3$	-H	$C_{26}H_{35}O_9$	491.2281	$-C_2H_4O_2$<br>$-C_2H_4O_2-H_2O$<br>$-C_2H_4O_2-2H_2O$<br>$-C_2H_4O_2-3H_2O$		
	lucibufagin E	-H	$-COCH_3$	$C_{26}H_{35}O_9$	491.2281			
	core lucibufagin	-H	-H	$C_{24}H_{33}O_8$	449.2175	$-H_2O$; $-2H_2O$<br>$-3H_2O$; $-4H_2O$		
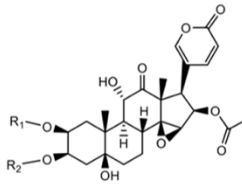	lucibufagin G	$-COCH_3$	$-COCH_3$	$C_{30}H_{37}O_{12}$	589.2285	$-C_6H_8O_5$<br>$-C_6H_8O_5-H_2O$<br>$- C_6H_8O_5-2H_2O$	$C_{24}H_{27}O_7$<br>$C_{24}H_{25}O_6$<br>$C_{24}H_{23}O_5$	427.1757<br>409.1651<br>391.1545
	lucibufagin H	$-COCH_3$	-H	$C_{28}H_{35}O_{11}$	547.2179	$-C_4H_6O_4-H_2O$<br>$-C_4H_6O_4-2H_2O$<br>$-C_4H_6O_4-3H_2O$		
	lucibufagin I	-H	$-COCH_3$	$C_{28}H_{35}O_{11}$	547.2179			
	lucibufagin J	-H	-H	$C_{26}H_{33}O_{10}$	505.2074	$-C_2H_3O_2-H_2O$<br>$-C_2H_3O_2-2H_2O$<br>$-C_2H_3O_2-3H_2O$		

Table S5. Screening MS^E mass data of each species for peaks matching the five LBGs common fragments.

Order	Species	Sample/Specimen info	# co-presence of 5 common fragments (known LBG #)
1	*Pyrocoelia rubrothorax*	Adult male	5 (2)
2	*Pyrocoelia praetexta*	Adult male	5 (2)
3	*Pyrocoelia pectoralis*	Adult male	2 (1)
4	*Pyrocoelia cenwanglaoensis*	Adult male	5 (2)
5	*Diaphanes lampyroides*	Adult male	6 (3)
6	*Diaphanes citrinus*	Adult male	5 (3)
7	*Aquatica leii*	Adult male	0
8	*Aquatica ficta*	Adult male	0
9	*Abscondita anceyi*	Adult male	0
10	*Curtos costipennis*	Adult male	0
11	*Sclerotia flavida*	Adult male	0
12	*Asymmetricata circmdata*	Adult male	0
13	*Emeia pseudosauteri*	Adult male	0
14	*Emeia pulchra*	Adult male	0
15	*Lamprigera sp.*	Larva	0
16	*Vesta sp.*	Adult male	0
17	*Rhagophthalmus sp. 2*	Adult female	0
18	*Lycocerus sp.*	Adult male	0
19	*Vesta* sp.2	Adult male	0
20	*Stenocladius* sp.	Adult male	0
21	*Cyphonocerus* sp.	Adult male	0

Table S6. Screening the MS^{E} mass data of each species for peaks matching the molecular weights with previously reported 29 LBGs.

Group	Species	LBGs Formula	$[M+H]^+$ theoretical	$[M+H]^+$ extract	Common Fragments #	MS/MS	Reference
		$C_{28}H_{36}O_{10}$	533.2386	533.2385	5	$[C_{28}H_{36}O_{10}+H]^+$	
		$C_{26}H_{34}O_{9}$	491.2281	491.2288	5	$[C_{26}H_{34}O_{9}+H]^+$	
	Pyrocoelia rubrothorax	$C_{24}H_{32}O_{8}$	449.2175	449.2177	5	$[C_{24}H_{32}O_{8}+H]^+$	
	Pyrocoelia praetexta	$C_{26}H_{32}O_{10}$	505.2074	505.2081	5	$[C_{26}H_{32}O_{10}+H]^+$	
	Pyrocoelia pectoralis	$C_{28}H_{36}O_{11}$	549.2335	549.2340	1*	N/A	(11, 12)
Group 1	*Pyrocoelia cenwanglaoensis*	$C_{28}H_{34}O_{10}$	531.2229	531.2236	5	N/A	
		$C_{28}H_{36}O_{9}$	517.2435	517.2437	5	N/A	
	Diaphanes lampyroides	$C_{28}H_{38}O_{10}$	535.2541	535.2538	5	N/A	
	Diaphanes citrinus	$C_{28}H_{28}O_{10}$	547.2540	547.2584	5	N/A	
		$C_{30}H_{40}O_{10}$	561.2694	561.2656	2*	N/A	
		Unknown	593.2568	593.2599	5	N/A	(13)
Group 2	*Aquatica leii*<br>*Aquatica ficta,*<br>*Abscondita anceyi*<br>*Curtos costipennis*<br>*Sclerotia flavida*<br>*Asymmetricata circmdata*<br>*Emeia pseudosauteri*<br>*Emeia pulchra*<br>*Lamprigera sp.*<br>*Vesta sp.*<br>*Rhagophthalmus sp. 2*<br>*Lycocerus sp.*<br>*Vesta* sp.2<br>*Stenocladius* sp.<br>*Cyphonocerus* sp.	$C_{29}H_{38}O_{10}$<br>$C_{30}H_{40}O_{10}$	547.2538<br>561.2694	547.2584<br>561.2574	0*<br>0*	N/A<br>N/A	(12, 14)

*Common fragments number less than five suggests the corresponding peak with matching molecular weight is not LBG.

Table S7. List of species that have been previously examined for LBGs.

Family/subfamily	Species	Sample information	Method	Produce LBGs?	Reference
Lampyridae/Lampyrinae	*Diaphanes lampyroides*	Larva	LC-MS	Yes	(15)
	Photinus marginellus	Adult male	NMR	Yes	(16)
	Photinus ignitus	Adult male	NMR	Yes	
	Photinus pyralis	Adult male, larva	LC-MS	Yes	(3)
	Ellychnia corrusca	Egg, larva, pupa, adult male & female	LC-MS+NMR	Yes	(11)
	Luciodota atra	Adult	HPLC	Yes	(17)
	Lampyris noctiluca	Larva, adult male & female	LC-MS	Yes	(12, 13)
	Pyractomena sinuata	Larva, adult	NA	Yes	(18)
	Lampyris algerica	Adult male & female	LC-MS	Yes	(13)
	Lampyris ambigena	Adult male	LC-MS	Yes	
	Lampyris angustula	Adult male	LC-MS	Yes	
	Lampyris brutia	Adult male	LC-MS	Yes	
	Lampyris germariensis	Adult male	LC-MS	Yes	
	Lampyris hellenica	Adult male	LC-MS	Yes	
	Lampyris lareynii	Adult male	LC-MS	Yes	
	Lampyris pallida	Adult male	LC-MS	Yes	
	Lampyris pseudozenkeri	Adult male	LC-MS	Yes	
	Lampyris raymondi	Adult male	LC-MS	Yes	
	Lampyris sariniae	Adult male	LC-MS	Yes	
	Lampyris zenkeri	Adult male	LC-MS	Yes	
	Nyctophila bonvouloirii	Adult male	LC-MS	Yes	
	Nyctophila heydeni	Adult male	LC-MS	Yes	
	Nyctophila libani	Adult male	LC-MS	Yes	
	Nyctophila maculicollis	Adult male	LC-MS	Yes	
	Nyctophila molesta	Adult male	LC-MS	Yes	
	Nyctophila reichii	Adult male	LC-MS	Yes	
	Pelania mauritanica	Adult male	LC-MS	Yes	
	Phosphaenus hemipterus	Adult male	LC-MS	Yes	
Lampyridae/Lamprohizinae	*Lamprohiza delarouzei*	Adult male	LC-MS	No	(13)
	Lamprohiza mulsantii	Adult male & female	LC-MS	No	
	Lamprohiza paulinoi	Adult male	LC-MS	No	
	Lamprohiza splendidula	Adult male & female	LC-MS	No	
	Lampyroidea achaiaca	Adult male	LC-MS	No	
	Lampyroidea dispar	Adult male	LC-MS	No	
	Lampyroidea syriaca	Adult male	LC-MS	No	
Lampyridae/Photurinae	*Photuris versicolor*	Adult female	HPLC	No	(17)
Lampyridae/Luciolinae	*Aquatica lateralis*	Larva, adult male	LC-MS+NMR	No	(3)
	Aquatica leii	Adult male	GC-MS	No	(19)
	Luciola italica	Adult male	LC-MS	No	(13)
	Luciola lusitanica	Adult male	LC-MS	No	
	Luciola novaki	Adult male	LC-MS	No	
Elateridae/Agrypninae	*Ignelater luminosus*	Adult male	LC-MS+NMR	No	(3)
Lycidae	*Dictyoptera aurora*	NA	LC-MS	No	(13)
	Erotides cosnardi	NA	LC-MS	No	
	Lopheros rubens	NA	LC-MS	No	
	Lygistopterus sanguineus	NA	LC-MS	No	
	Platycis minutus	NA	LC-MS	No	
	Pyropterus nigroruber	NA	LC-MS	No	

Table S8. Three fossil calibrations used (A) and divergence time estimates with comparison to previous studies (B). Posterior means and 95% intervals (in parentheses) are in Mya.

A

Fossil	Age (Mya)	Clade
Protoluciola albertalleni(20)	100	Luciolinae
Cretophengodes azari(21)	99	Phengodidae + Rhagophthalmidae
Litholacon, *Ageratus* and *Cryptocardius*(22, 23)	166.1–157.3	Elateridae

B

Node	This study	Previous studies		
	MCMCTree	(24)	(25)	(7)
MRCA of Lampyrinae	66 (56-76)	59 (53-70)	88 (70-103)	48 (30.29-66.27)
Lampyrinae and Photurinae	93 (81-107)	61 (55-72)	106 (90-127)	N/A
MRCA of Lampyridae	136 (122-153)	133.18 (117.86-152.47)	139.85 (108.43-165.68)	100 (74.38-129.33)

Table S9. Hydrogen bonds formed between bufalin or core lucibufagin with the pig ATP1A1 (PDB 4RES).

Interactions sites[1]	Known effect on cardiac glycosides sensitivity?	Hydrogen bonds formed?		Distance between H-bond donor and accepter	
		Bufalin	Core lucibufagin	Bufalin	Core lucibufagin
Q111	Yes	No	No	N/A	N/A
E117	No	No	Yes	N/A	2.8
D121	Yes	Yes	No	3.3	N/A
N122	Yes	No	Yes (2 H-bonds)	N/A	3.1/3.5
V322	No	Yes	No	3.4	N/A
E327	Yes	Yes	Yes	2.9	3.5
T797	Yes	Yes	Yes	2.8	3.3

[1] The positions are numbered according to the mature pig enzyme

Table S10. Docking simulations of core lucibufagin to native ATPα proteins from firefly species.

Native protein	LBGs presence	RMSD[1] from pig ATP1A1 docking position (Å)	Docking score (Affinity)
Diaphanes lampyroides	Yes	0.892	-6.7782
Pyrocoelia analis	Yes	1.230	-5.8252
Pyrocoelia pectoralis	Yes	1.230	-5.7382
Pyrocoelia rubrothorax	Yes	1.259	-6.5962
Photinus pyralis	Yes	1.281	-6.5947
Photinus australis	Yes	0.899	-6.4763
Photinus scintillans	Yes	1.326	-6.7432
Photinus carolinus	Yes	1.316	-6.9404
Photinus macdermotti	Yes	0.873	-6.7090
Lucidota atra	Yes	0.790	-6.2943
average	-	-	-6.4696
Lamprigera sp.	No	0.929	-7.8950
Asymmetricata circumdata	No	0.794	-7.7342
Abscondita sp.	No	0.996	-8.0570
Curtos costipennis	No	0.737	-7.6765
Aquatica lateralis	No	1.284	-7.0120
Aquatica ficta	No	0.594	-7.3704
Aquatica leii	No	1.312	-7.7970
Sclerotia flavida	No	1.301	-7.7336
Luciola aquatilis	No	1.257	-7.7722
average	-	-	-7.6720

[1]RMSD between the best structure of core lucibufagin docking to the native ATPα proteins (the 'best' structure was defined as the structure from the top 5 highest affinity dockings that was closest to the co-crystal coordinates of core lucibufagin binding to pig ATP1A1.

Table S11. Docking simulations of core lucibufagin onto pig ATP1A1 carrying specific amino acid substitution

Substitution(s)	Known effect on cardiac glycosides sensitivity	RMSD from best[1] WT docking position (Å)	Docking score (Affinity)	Contacts formed by the β-surface of the steroid core (<4Å)[2]
A101S	No	1.085	-6.5823	LBG-Q111<br>LBG-E117<br>LBG-A323<br>LBG-E779<br>LBG-D804
A113T	No	1.206	-6.8257	LBG-A111<br>LBG-N122<br>LBG-T797
Q119I	Yes	0.944	-5.6707	LBG-Q119<br>LBG-N122<br>LBG-E327<br>LBG-T797
T861A	No	1.295	-6.6178	LBG-E117<br>LBG-N122<br>LBG-A323
I874P	Yes	1.125	-6.4923	LBG-N122<br>LBG-V322<br>LBG-E327<br>LBG-T797
A101S+<br>A113T+<br>Q119I+<br>T861A+<br>I874P	-	1.597	-6.0266	LBG-Q111<br>LBG-E117<br>LBG-N122<br>LBG-E327<br>LBG-T797
None (WT)	-	-	-7.8552	LBG-E117<br>LBG-N122(2)<br>LBG-E327<br>LBG-T797

[1]The 'best' WT docking of core lucibufagin was defined as the structure in the top 10 highest affinity dockings that was closest to the co-crystal coordinates of bufalin.
[2]In addition to the listed interactions formed by the β-surface of the steroid core and residues in αM1-M2 and αM4 of ATP1A1, there are a number of van der Waals interactions between the α-surface of the steroid core and residues in αM5-M6. These interactions are not listed separately as they were mostly unaffected by the substitutions.

SI References

1. Fu X *et al.* 2017. Long-read sequence assembly of the firefly *Pyrocoelia pectoralis* genome. *Gigascience.* 6:1-7.
2. Sander SE, Hall DW. 2015. Variation in opsin genes correlates with signalling ecology in North American fireflies. *Mol. Ecol.* 24:4679-4696.
3. Fallon TR *et al.* 2018. Firefly genomes illuminate parallel origins of bioluminescence in beetles. *Elife.* 7:1-146.
4. Martin GJ, Lower SE, Suvorov A, Bybee SM. 2021. Molecular Evolution of Phototransduction Pathway Genes in Nocturnal and Diurnal Fireflies (Coleoptera: Lampyridae). *Insects.* 12:561.
5. Amaral DT, Silva JR, Viviani VR. 2017. Transcriptomes from the photogenic and non-photogenetic tissues and life stages of the *Aspisoma lineatum* firefly (Coleoptera: Lampyridae): Implications for the evolutionary origins of bioluminescence and its associated light organs. *Gene Rep.* 8:150-159.
6. McKenna DD *et al.* 2019. The evolution and genomic basis of beetle diversity. *Proc Natl Acad Sci USA.* 116:24729-24737.
7. Zhang R *et al.* 2020. Genomic and experimental data provide new insights into luciferin biosynthesis and bioluminescence evolution in fireflies. *Sci. Rep.* 10:15882.
8. Vongsangnak W, Chumnanpuen P, Sriboonlert A. 2016. Transcriptome analysis reveals candidate genes involved in luciferin metabolism in *Luciola aquatilis* (Coleoptera: Lampyridae). *PeerJ.* 4:e2534.
9. Amaral DT, Silva JR, Viviani VR. 2017. Transcriptional comparison of the photogenic and non-photogenic tissues of *Phrixothrix hirtus* (Coleoptera: Phengodidae) and non-luminescent *Chauliognathus flavipes* (Coleoptera: Cantharidae) give insights on the origin of lanterns in railroad worms. *Gene Rep.* 7:78-86.
10. Wang K, Hong W, Jiao H, Zhao H. 2017. Transcriptome sequencing and phylogenetic analysis of four species of luminescent beetles. *Sci. Rep.* 7:1814.
11. Smedley SR *et al.* 2017. Bufadienolides (lucibufagins) from an ecologically aberrant firefly (*Ellychnia corrusca*). *Chemoecology.* 27:141-153.
12. Tyler J, Mckinnon W, Lord GA, Hilton PJ. 2008. A defensive steroidal pyrone in the glow-worm *Lampyris noctiluca* L. (Coleoptera : Lampyridae). *Physiol. Entomol.* 33:167-170.
13. Berger A, Petschenka G, Degenkolb T, Geisthardt M, Vilcinskas A. 2021. Insect Collections as an Untapped Source of Bioactive Compounds—Fireflies (Coleoptera: Lampyridae) and Cardiotonic Steroids as a Proof of Concept. *Insects.* 12:689.
14. Rawlinson C *et al.* 2020. Hierarchical clustering of MS/MS spectra from the firefly metabolome identifies new lucibufagin compounds. *Sci. Rep.* 10:1-9.
15. Yoshida T *et al.* 2020. Dramatic dietary shift maintains sequestered toxins in chemically defended snakes. *Proc Natl Acad Sci USA.* 117:5964-5969.
16. Eisner T, Wiemer DF, Haynes LW, Meinwald J. 1978. Lucibufagins: Defensive steroids from the fireflies *Photinus ignitus* and *P. marginellus* (Coleoptera: Lampyridae). *Proc Natl Acad Sci USA.* 75:905-908.

17. Gronquist M *et al.* 2006. Shunning the night to elude the hunter: diurnal fireflies and the "femmes fatales". *Chemoecology.* 16:39-43.
18. Tonyai K, Farrar, M., & Robertson, H. . 2017. A multi-faceted investigation of the biology of *Pyractomena sinuta*, a rare firefly. *Twelfth annual summer research symposium trinity college.*
19. Fu X *et al.* 2007. Structure and function of the eversible glands of the aquatic firefly *Luciola leii* (Coleoptera: Lampyridae). *Chemoecology.* 17:117-124.
20. Kazantsev S. 2015. *Protoluciola albertalleni* gen. n., sp. n., a new Luciolinae firefly (Insecta: Coleoptera: Lampyridae) from Burmite amber. *Russ. Entomol. J.* 24:281-283.
21. Li Y *et al.* 2021. Cretophengodidae, a new Cretaceous beetle family, sheds light on the evolution of bioluminescence. *Proc. R. Soc. B.* 288:20202730.
22. Dolin V. 1980. Click-beetles (Coleoptera, Elateridae) from Upper Jurrasic of Karatau. *Mesozoic Fossil insects. Naukova Dumka Publ House, Kiev.* 17-81.
23. Kundrata R, Packova G, Prosvirov AS, Hoffmannova J. 2021. The fossil record of Elateridae (Coleoptera: Elateroidea): Described species, current problems and future prospects. *Insects.* 12:286.
24. Powell GS *et al.* 2021. Firefly bioluminescence outshines aerial predators. *bioRxiv.*
25. Höhna S, Lower SE, Duchen P, Catalán A. 2021. A Time-calibrated Firefly (Coleoptera: Lampyridae) Phylogeny: Using Genomic Data for Divergence Time Estimation. *bioRxiv.*
